# Inhibition of Histone Lysine Acetyltransferases by Coenzyme A Analogs

**DOI:** 10.3390/molecules31030477

**Published:** 2026-01-29

**Authors:** Faidra Voukia, Nurgül Bilgin, Steffen Bundgaard Andersen, Jasmin Mecinović

**Affiliations:** Department of Physics, Chemistry and Pharmacy, University of Southern Denmark, Campusvej 55, 5230 Odense, Denmark; phaev93@hotmail.com (F.V.); bilgin@sdu.dk (N.B.); steffen.bundgaard.andersen@gmail.com (S.B.A.)

**Keywords:** histone, epigenetics, lysine acetyltransferases, acetylation, coenzyme A

## Abstract

Histone lysine acetylation is a widespread posttranslational modification, essential for vital functions in eukaryotic organisms. Histone lysine acetyltransferases (KATs) employ acetyl-coenzyme A as a universal acetyl donor for acetylation of lysine residues in histone and non-histone proteins. Despite the biomedicinal importance of modulation of the KAT activity, application of the acetyl-coenzyme A cosubstrate structure for the design of potent and selective inhibitors has been underexplored. Here, we developed functionalized coenzyme A analogs as inhibitors against human histone lysine acetyltransferases GCN5, KAT8, and HAT1. In contrast to the unmodified coenzyme A, which was found to be a poor inhibitor of GCN5 and KAT8 (IC_50_ > 150 μM), we showed that a ketone-substituted coenzyme A was the most potent inhibitor of GCN5 and KAT8 (IC_50_ = 10.9 μΜ and 13.6 μΜ, respectively). Coenzyme A and an acetamide-substituted coenzyme A efficiently inhibited HAT1 (IC_50_ = 7.3 μΜ and IC_50_ = 3.9 μΜ, respectively). Our work demonstrates that human KATs can be efficiently and selectively inhibited by S-functionalized coenzyme A, the results exhibiting significant potential towards development of highly active chemical probes for biomedically important KATs.

## 1. Introduction

**Figure 1:**
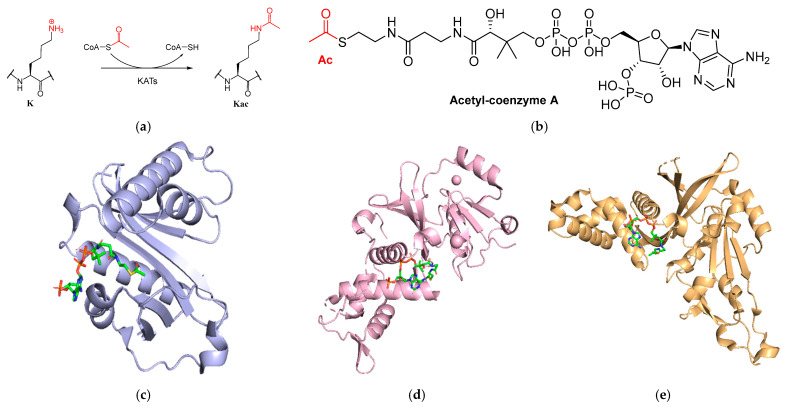
(**a**) Acetylation of lysine (K) by AcCoA, under KAT catalysis, yielding CoASH and acetyl-lysine (Kac). (**b**) Structure of AcCoA, with the acetyl group (Ac) highlighted in red. (**c**) View on the crystal structure of AcCoA (green) bound to human GCN5 (blue) (PDB: 1Z4R). (**d**) View on the crystal structure of AcCoA (green) bound to human KAT8 (pink) (PDB: 2GIV). (**e**) View on the crystal structure of AcCoA (green) bound to human HAT1 (yellow) (PDB: 2P0W).

Proteins are subjected to various posttranslational modifications (PTMs), which are essential to the fine-tuning of protein function. Lysine acetylation is a widespread posttranslational modification on proteins and generally an omnipresent reaction in eukaryotes [1,2,3]. This reaction is carried out by lysine acetyltransferases (KATs), which use acetyl-coenzyme A (AcCoA) as the acetyl group donor, yielding the acetylated lysine product, as well as coenzyme A (CoASH) as a byproduct (Figure 1a,b) [4,5,6,7,8]. The mechanism of enzymatic acetylation entails a deprotonation of the lysine substrate, after which it can perform a nucleophilic attack on the electrophilic acetyl group of AcCoA [3].

In proteins, known substrates of acetyltransferases include the N-terminus of the protein, which is the most commonly occurring acetylation [9,10,11], the Nε-position of the lysine side chain [11,12,13,14], and tyrosine/serine/threonine at the OH group in the side chain [11,14]. Lysine acetylation catalyzed by KATs is a thoroughly studied PTM, especially on histones, since it is vital for epigenetic transcription regulation and DNA repair [15,16,17,18]. Upon acetylation of lysine in histones, the positive charge of histones is neutralized, altering the affinity of histones with DNA and thus affecting the accessibility of a gene for transcription or repair [2,3,19,20]. The three main families of KATs responsible for histone lysine acetylation include GNAT, p300/CBP, and MYST. Members of the GNAT family share very similar modes for AcCoA binding, implying the existence of a conserved mechanism. Generally, the AcCoA binding pocket is located between the β4 and β5 strands of these enzymes. AcCoA binds in a sharply bent conformation, as a result of two hydrogen bonds to the β4 main-chain groups, one being with the acetyl group of AcCoA [21]. In the proposed mechanism, the acetyl group is transferred directly from AcCoA to the lysine substrate via direct nucleophilic attack by the lysine’s ε-amino group on the acyl-carbon, forming a ternary intermediate during this process [21,22]. In the case of the p300/CBP family, named after the two human paralogs p300 and CBP [23], a Theorell−Chance mechanism appears to be involved. In this mechanism, there is no ternary intermediate. Instead, the substrate associates very briefly with the enzyme, separating immediately after the reaction [24,25]. The AcCoA binding region in p300/CBP appears to be highly conserved and shows similarities to KATs of other families, such as GCN5 and Esa1 [26]. Here, AcCoA is surrounded by several overlapping β-strands and α-helices, similarly to GCN5 of the GNAT family [27]. However, it also features a specialized loop, which forces the entire structure of the lysine substrate and the AcCoA cosubstrate to adopt even more rigid formation [24,28]. Finally, the MYST members of KATs contain the characteristic MYST domain, which contains a zinc finger and an AcCoA binding site homologous to the binding site in GNAT KATs [29,30]. This AcCoA binding site contains highly conserved cysteine and glutamate residues that are crucial to their catalytic mechanism [29,31,32]. This is referred to as a ping-pong mechanism, where an acetylated intermediate is formed with Cys304 and Glu338 acts as a base, required for the formation of the acetylated lysine [23,29,33].

KATs are enzymes of high biomedical importance, and aberrant function of KATs has been linked with multiple diseases. Mutations in certain histone KATs, which is the main cause of these dysregulations, have been involved in different cancers, such as in the lung [34], breast [35], prostate [36], as well hepatocellular carcinoma [37], and outside of cancers, Alzheimer’s disease [38]. Therefore, it has been established that KATs are significant biomedical targets, making it important to design and develop potent and selective inhibitors for KATs implicated in disease [39,40,41,42,43].

To date, there have been different strategies for inhibition of KATs. First, inhibitors of KATs have been synthesized based on heterocycles, such as thiazoles [44,45,46,47], isothiazoles [48,49,50,51], and other nitrogenous heterocycles [52,53,54]. The second strategy involves natural products and their derivatives. This strategy has been especially effective against the p300/CBP family of KATs and involves analogs of, for example, anacardic acid [55,56,57], quinolines [58,59], or catechin [60,61,62]. The final strategy is the development of bisubstrate inhibitors, which contain two different moieties connected with a linker. These include a CoASH moiety, which binds to the AcCoA binding site, and a substrate-mimicking moiety, which can be an organic molecule or an oligopeptide [39]. This structure aids in increasing selectivity and potency. Examples of enzymes that have been successfully and selectively inhibited (in micromolar to nanomolar range) include p300 [63,64], PCAF, GCN5 [27,64], Esa1, Tip60 [65], KAT8 [66,67], and HAT1 [68].

In this work, a selection of S-functionalized CoASH analogs with diverse small organic structures was designed and synthesized. These organic structures are smaller than the previously reported bisubstrate inhibitors and can be synthesized in a facile manner. These conjugates were subsequently tested against three human histone KATs: GCN5 (also known as KAT2A) from the GNAT family, KAT8 (also called MOF) from the MYST family, and HAT1 (also known as KAT1) from the GNAT family (Figure 1c–e).

## 2. Results

### 2.1. Chemistry

**Scheme 1:**
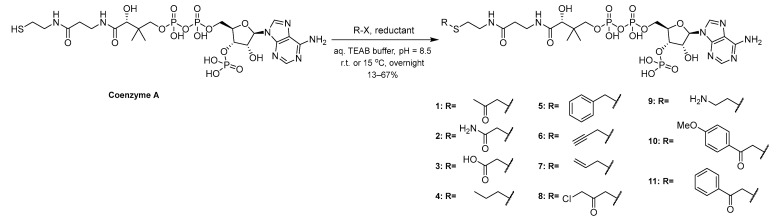
Synthesis of CoASH analogs.

A selection of CoASH analogs with diverse small organic structures was designed and synthesized (Scheme 1). These structures bear similarities to previously reported bisubstrate inhibitors of KATs, in that they involve CoASH analogs with substrate mimicking moieties and have demonstrated that CoASH analogs are able to inhibit KATs [39]. We chose small analog structures that contain diverse functionalities of varied sizes in an attempt to explore the AcCoA binding pocket of KATs. Compounds **1**–**3** and **8** consist of acetylated structures of differing chemical properties; a ketone, an amide, a carboxylic acid, and a chloroacetyl group, respectively. Compounds **4**, **6**, and **7** contain alkyl groups of different rigidities, i.e., propyl, propargyl, and allyl substitution, respectively, whereas compound **5** contains a bulkier benzyl group. Compound **9** involves a polar ethylamine-substitution. Apart from its interest due to the basic functionality, we also envisioned that it could be further functionalized by imine formation in the future. Compounds **10** and **11** carry bulky acetophenone substitutions. Furthermore, **10** carries an electron-donating methoxy group. Briefly, the compounds were synthesized from CoASH with the corresponding halides (chloroacetone, chloroacetamide, bromoacetic acid, iodopropane, benzyl-chloride, propargyl chloride, allylchloride, 1,3-dichloroacetone, 2-bromoethylamine hydrobromide, 4-methoxy phenacetyl bromide, and phenacetyl bromide, respectively, for **1**–**11**) in aqueous triethylammonium bicarbonate (TEAB) in varying yields. A reductant (DTT, or TCEP for **9**) was also added to prevent CoASH disulfide formation.

### 2.2. Biological Evaluation

MALDI-TOF MS-based assays were carried out to determine the ability of the synthesized CoASH analogs to inhibit the activity of recombinantly expressed human histone acetyltransferases GCN5, KAT8, and HAT1, three biomedically important epigenetic enzymes. GCN5 catalyzes the acetylation of histone H3K9/14/18/23, KAT8 catalyzes the acetylation of histone H4K5/8/16, and HAT1 catalyzes the acetylation of histone H4K5 and H4K12 [1,39,69,70]. In this work, we focused on the acetylation of H3K9, H4K16, and H4K12 peptides by GCN5, KAT8, and HAT1, respectively. The CoASH analogs were investigated in parallel with the CoASH control, as it is the natural by-product of the acetyl-CoA-dependent acetylation reaction. In these assays, we used previously reported conditions to properly assess the competitivity of the inhibitors against acetyl-CoA, with histone peptides carrying the acetylation site of interest being used as substrates (see Section 4).

**Figure 2:**
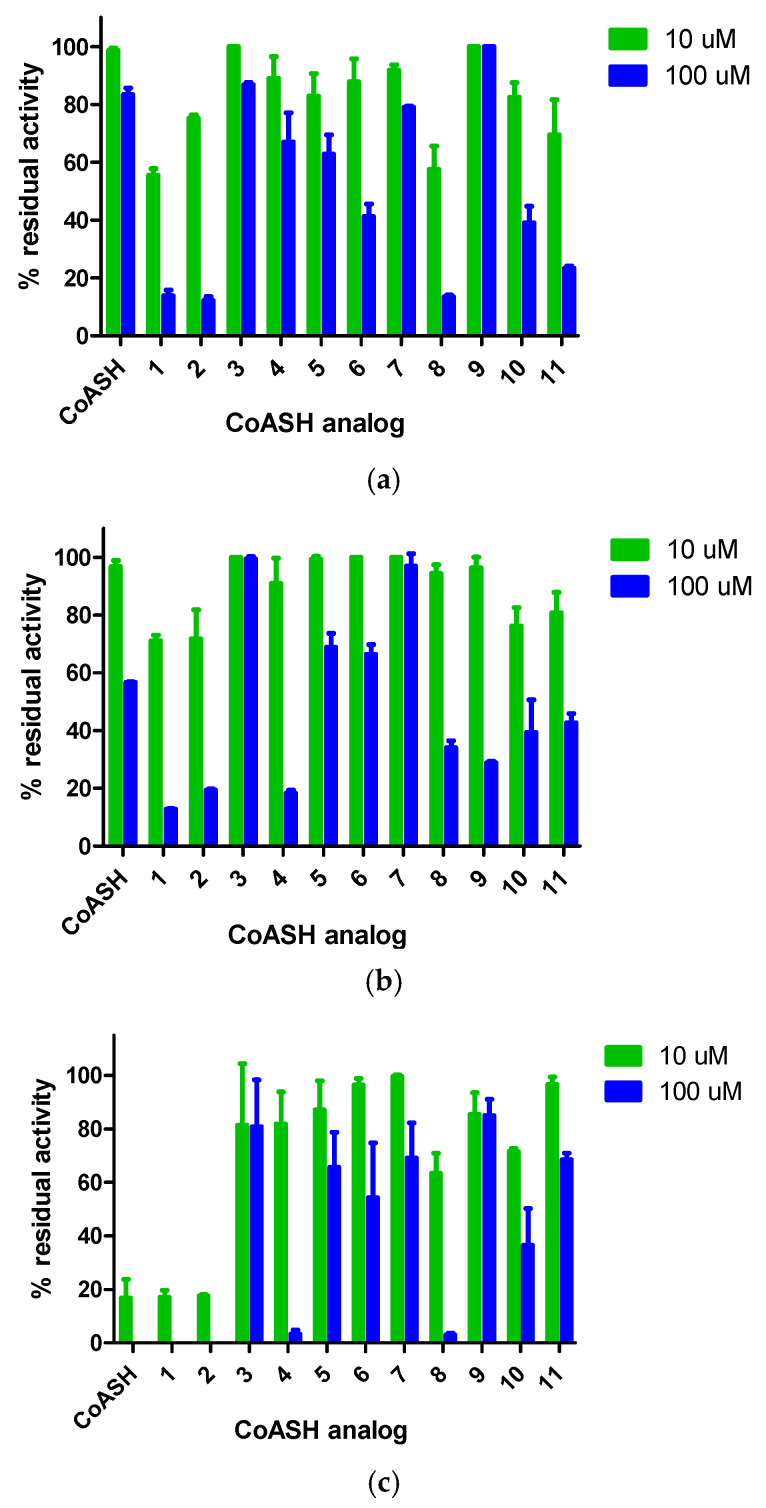
Single point inhibition assays of CoASH and CoASH analogs with (**a**) GCN5, (**b**) KAT8, and (**c**) HAT1.

**Figure 3:**
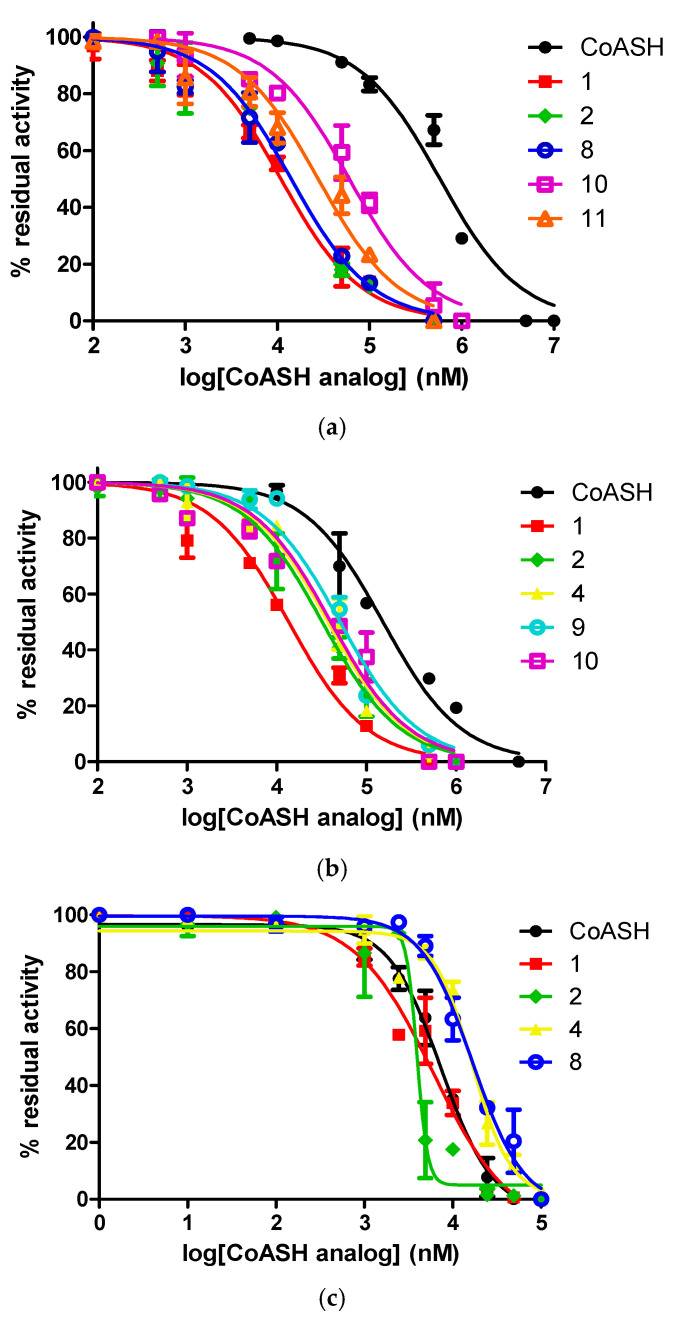
Dose–response curves for the most potent inhibitors of (**a**) GCN5, (**b**) KAT8, (**c**) HAT1, in comparison to CoASH.

Initially, single point inhibition assays at 10 and 100 μM of all analog compounds were performed to probe their potential inhibitory activity against each acetyltransferase (Figure 2). In the case of GCN5 and KAT8, most of the CoASH analogs evaluated were better inhibitors in comparison to CoASH. The results for GCN5 pointed towards compounds **1**, **2**, **6**, **8**, **10**, and **11** having an IC_50_ value of <100 μM, while the same was observed with compounds **1**, **2**, **4**, **8**, **9**, **10**, and **11** as KAT8 inhibitors. In the case of HAT1, CoASH was significantly a better inhibitor, but compounds **1**, **2**, **4**, and **8** were also expected to have an IC_50_ value < 100 μM. Subsequently, the IC_50_ values were measured for all potent compounds against the three KAT enzymes. The dose–response curves are also shown (Table 1, Figure 3 and Figures S1–S3).

For GCN5, CoASH seems to be a very poor inhibitor (IC_50_ = 581 μM), and most of the CoASH analogs that were evaluated performed better in comparison. The most potent inhibitor was ketone-substituted **1** (IC_50_ = 10.9 μM, more than 50-fold increased potency), followed closely by acetamide-substituted **2** and chloroacetone-substituted **8** (IC_50_ = 14.1 μM for both) (Table 1, Figure 3a and Figure S1). Propargyl-substituted **6**, methoxyphenacetyl-substituted **10**, and phenacetyl-substituted **11** were the other three compounds displaying poor inhibition (IC_50_ = 85.0, 60.4 and 27.7 μM, respectively) (Table 1, Figure 3a and Figure S1). It is also noteworthy that the three most potent compounds contained a carbonyl group—as well as the observation that phenacetyl-substituted **10** and **11**, though not the most potent, performed markedly better than benzyl-substituted **5** (IC_50_ >100 μM), which lacked a carbonyl group (Table 1, Figure S1).

For KAT8, CoASH seems to be more potent than for GCN5 (IC_50_ = 153 μM); however, most of the CoASH analogs performed better in comparison. The most potent inhibitor was ketone-substituted **1** (IC_50_ = 13.8 μM, about 11-fold increased potency), followed by acetamide substituted **2** and propyl-substituted **4** (IC_50_ = 32.2 and 37.4 μM, respectively). Chloroacetone-substituted **8**, ethylamino-substituted **9**, methoxy-phenacetyl-substituted **10**, and phenacetyl-substituted **11** were the other four compounds displaying IC_50_ values lower than 100 μM (IC_50_ = 70.5, 50.8, 40.1, and 63.9 μM, respectively) (Table 1, Figure 3b and Figure S2). Remarkably analog **9**, which exhibited no inhibition towards GCN5, was more active against KAT8, with an IC_50_ of 50.8 μM. Furthermore, even though we observed increased potency with compounds lacking a carbonyl group, once again, phenylcarbonyl-substituted **10** and **11** were more potent than carbonyl-free benzyl-substituted **5** (IC_50_ >100 μM) (Figure 3b and Figure S2).

The inhibitory activity of selected analogs was significantly better against HAT1, where CoASH exhibited the highest inhibitory activity (IC_50_ = 7.3 μM) in comparison with the other two tested enzymes, and most of the CoASH analogs displayed lower activity in comparison (Table 1, Figure 3c and Figure S3). The analogs displaying more potent inhibition than CoASH were ketone-substituted **1** and acetamide-substituted **2** (IC_50_ = 5.8 and 3.9 μM, respectively), maintaining the pattern of relatively high inhibitory activity. Propyl-substituted **4** and chloroacetone-substituted **8** also displayed IC_50_ values lower than 100 μM (IC_50_ = 15.7 and 16.6 μM, respectively) (Figure 3c and Figure S3).

### 2.3. Molecular Docking

**Figure 4:**
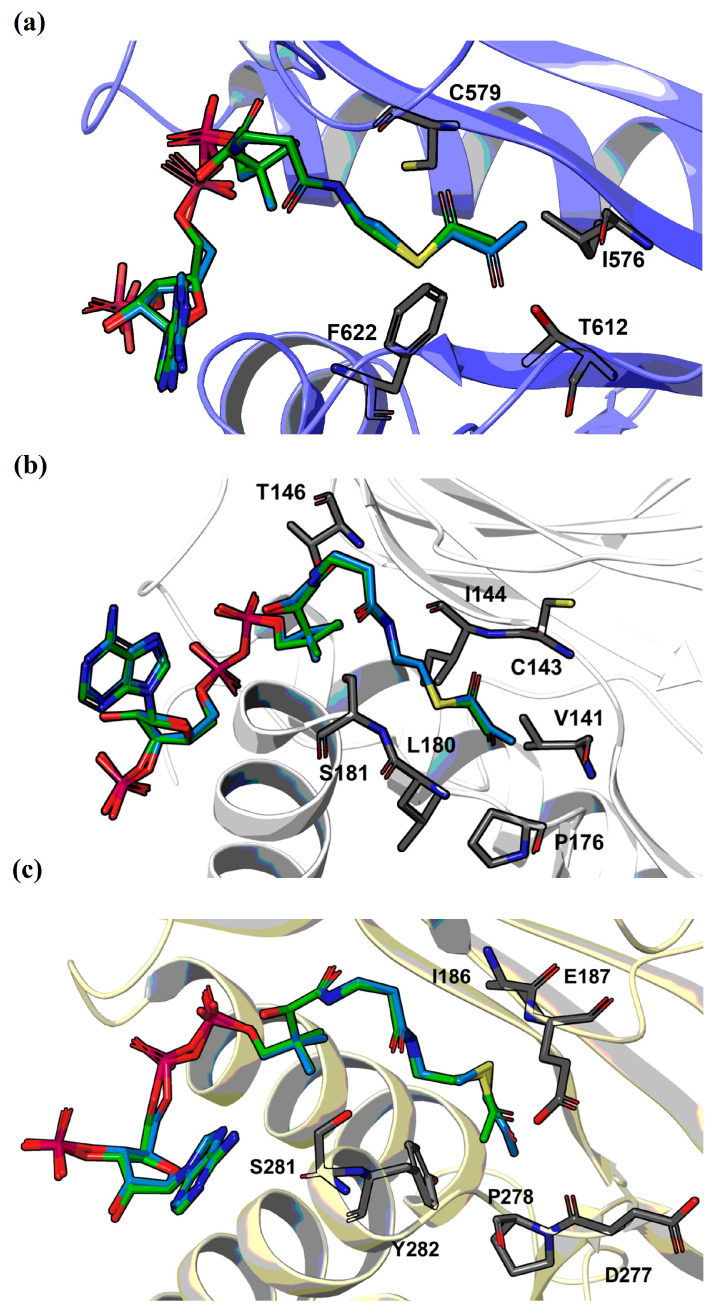
Molecular docking poses comparing AcCoA (green) and analog **1** (blue). (**a**) Superimposed docking poses of AcCoA (green) and **1** (blue) with GCN5 (purple, PDB: 1Z4R). (**b**) Superimposed docking poses of AcCoA (green) and **1** (blue) with KAT8 (white, PDB: 2GIV). (**c**) Superimposed docking poses of AcCoA (green) and **1** (blue) with HAT1 (wheat, PDB: 2P0W).

Molecular docking was then carried out to predict the binding pose of the representative, very potent CoASH-based inhibitor, compound **1**, of the tested GCN5, KAT8, and HAT1 enzymes in comparison with acetyl-CoA. Our docking results show that the active site of all three enzymes can well accommodate inhibitor **1**, and that the ketone moiety aligns well with the corresponding acetyl moiety of acetyl-CoA (Figure 4). Most of the interactions between the individual enzymes and acetyl-CoA in the complex structure are still conserved with inhibitor **1**. The molecular docking predicted that the ketone moiety of inhibitor **1** could interact with Ile576 and Phe622 of GCN5 through hydrophobic contacts and further stabilized by H-bonding interaction with Thr612 (Figure 4a). In the case of binding to KAT8, inhibitor **1** could be involved in hydrophobic interactions through its ketone moiety with residues Val141 and Pro176; however, the H-bonding interaction with Ile144 backbone appears to be disrupted (Figure 4b). Binding of inhibitor **1** to HAT1 is governed by hydrophobic interactions of the ketone moiety with residues Ile186 and Tyr282 (Figure 4c). It is worth noting that the acetyl group of acetyl-CoA is deeply buried within the active site of GCN5, KAT8, and HAT1, restricting access for bulky substituents and supporting our observations for poor inhibition of bulkier CoA derivatives. Consequently, elongated acyl chains require a degree of conformational flexibility to be accommodated within the binding pocket without steric clashes, allowing them to adapt to the active site.

## 3. Discussion

The similarities and differences in the activities of the assessed S-functionalized coenzyme A derivatives between GCN5, KAT8, and HAT1 could be traced back to the structure of the binding pocket and what is known about the catalytic mechanism of these acetyltransferases. Despite the proposal that the MYST and GNAT family acetyltransferases operate on different mechanisms, both KAT8 and GCN5 possess a conserved cysteine residue in the AcCoA binding pocket. In the case of KAT8, it is Cys143 [29], while in GCN5, it is Cys579 [22], according to the known crystal structures of the enzymes. In both cases, those cysteines form hydrogen bonds with the carbonyl oxygen of the acetyl group, and in the case of KAT8, it is suggested that Cys143 actively takes a part in the enzyme’s catalytic mechanism [29]. Therefore, this can be connected to the beneficial effect that the presence of carbonyl groups appears to have on the inhibitory activity of most carbonyl-possessing CoASH analogs. On the other hand, it can explain the low inhibitory activity of CoASH: the loss of the acetyl group from AcCoA eliminates its hydrogen bond with the cysteine residue in the binding pocket of GCN5 and KAT8, decreasing the binding affinity. In the case of HAT1, there is still lacking knowledge surrounding the catalytic mechanism of the enzyme. It has been suggested that Glu187 and Glu276 could participate in the acetylation reaction. Furthermore, in the binding site of AcCoA, no hydrogen bonds are observed between the acetyl group and surrounding residues, with residues Ile186, Pro278, and Tyr282 forming hydrophobic interactions instead [71]. This could provide an explanation for the higher tolerance of the enzyme towards the more active hydrophobic CoASH analogs, while structure rigidity and sterical hindrance may be attributed to the lowered activity of most of the analogs. Furthermore, this suggests that the lack of the acetyl group in CoASH does not have a significant effect on its affinity for the binding pocket, resulting in a higher inhibitory activity for CoASH, especially compared to some of the bulkier, hydrophobic CoASH analogs.

As for the differences in selectivity for specific compounds, acetamide **2** and chloroacetylated **8** were more selective towards GCN5 and HAT1, propylated **4** was selective towards KAT8 and HAT1, ethylaminated **9** was selective towards KAT8, and unmodified coenzyme A was selective towards HAT1. These results point towards structural nuances in the binding pockets of the two acetyltransferases, where the size, rigidity, and polarity of the substituent play a part in the tolerance of the CoASH analog by the enzyme.

In line with our experimental results, molecular docking analyses suggest that the ketone-substituted CoASH (**1**) is well accommodated by all three enzymes, supporting its efficient inhibitory activity. It is worth noting that GCN5, KAT8, HAT1, and other KATs are reported to carry out related acylation reactions, transferring acyl groups bulkier than acetyl, such as propionyl, butyryl, crotonyl, benzoyl, and many others [72,73]. Each enzyme has comparable selectivity for employing propionyl-CoA [74,75,76], and therefore, it is not surprising that inhibitor **1**, which is most similar to propionyl-CoA with the only difference being the position of the carbonyl group, can be utilized and hereby inhibit all three enzymes. This is also supported by the superimposed structures of inhibitor **1** and propionyl-CoA with GCN5 and KAT8 (Figure S4). However, the extended and bulkier substitutions, most similar to butyryl-CoA and benzoyl-CoA, are not tolerated well by the enzymes, which is also reflected by their preferred selectivity for acetyl-CoA [76,77].

To our knowledge, there have not been extensive reports of CoASH-based bisubstrate inhibitors targeting KAT8, GCN5, and HAT1. These bisubstrates involve more complex structures than the ones presented in this work, consisting of the CoASH structure linked to a longer histone substrate peptidomimetic rather than a simple substituent. In one work, four such compounds were reported for GCN5, with H3-CoA-20 and H3-(Me)CoA-20 being the most potent (IC_50_ = 0.5 and 0.3 μM, respectively) [27]. HAT1 was targeted with a similar peptidomimetic, H4K12-CoA (Ki value of 1.1 nM) [78]. On the other hand, inhibitors of KAT8 that have been reported so far belong to a class of small organic molecule inhibitors [66,67]. When comparing these reports to the eleven analogs screened in our work, especially inhibitors **1** and **2**, it is observed that smaller substituents were still able to result in efficient inhibition of GCN5 and HAT1. Smaller substituents bring the added advantage of a more facile synthetic procedure, making further optimizations towards nM scale inhibitors more accessible compared to structures incorporating peptidomimetics. Finally, our results for KAT8 suggest that there is potential in further researching the bisubstrate approach to its inhibition, which has been so far unexplored.

## 4. Materials and Methods

### 4.1. General Experimental Procedures

^1^H and ^31^P NMR spectra were recorded using a Bruker Avance III 400 MHz NMR spectrometer (Fällanden, Switzerland) and a JEOL JNM-ECZR 500 MHz NMR spectrometer (Kobe, Japan). Chemical shifts are reported in parts per million (ppm δ) referenced to the residual ^1^H resonance and the residual ^31^P resonance of the deuterated solvent. Splitting patterns are designated as follows: s, singlet; d, doublet; dd, doublet of doublets; ddd, doublet of doublet of doublets; t, triplet; td, triplet of doublet; q, quartet; and m, multiplet. Coupling constants (J) are reported in hertz (Hz). MALDI-TOF spectra were measured in a Bruker Daltonics UltrafleXtreme-II tandem mass spectrometer (Bremen, Germany). High-resolution mass spectra (HRMS) were recorded on a Bruker Daltonics-micrOTOF-Q II-ESI-Qq-TOF mass spectrometer (Copenhagen, Denmark). Analytical RP-HPLC employed a Gemini 5 mm C18 110 LC column (Phenomenex) at a flow rate of 1 mL/min with a gradient of H_2_O + 0.1% TFA and ACN + 0.1% TFA from 1 (*v*/*v*) for 1 min to 100% ACN + 0.1% (*v*/*v*) TFA at 11 to 15 min and back to 10 from 16 to 20 min. Analytical spectra were monitored at 254 nm. All reagents and solvents were purchased from commercial sources and used without further purification.

### 4.2. Synthesis of CoASH Analogs

#### 4.2.1. General Procedure for Synthesis of **1**–**11**

First, 1 eq. of coenzyme A was dissolved in 150–600 μL TEAB (50 mM) buffer, pH = 8.5 under argon, which also contained 1M DTT (or TCEP for **9**) that was freshly added. The solution was stirred in a thermomixer at 15 °C (**1**–**8**) or r.t. (**9**–**11**), at 1000 rpm for 1 h, and then 2 eq. of the appropriate halide (chloroacetone, bromo-acetic acid, propargyl chloride, chloroacetamide, benzyl-chloride, iodopropane, allylchloride, 1,3-dichloroacetone, 2-bromoethylamine hydrobromide, 4-methoxyphenylacetyl bromide, or phenylacetyl bromide) were added to the solution. The mixture was stirred at 15 ^°^C (**1**–**8**) or r.t. (**9**–**11**) overnight, after which it was purified through reverse-phase preparative HPLC (RP-HPLC) using a linear gradient of H_2_O + 0.2% TFA and MeOH from 0 to 100% MeOH over 90 min at 3 mL/min and a Gemini 10 µm NX-C18 110 Å LC column (Phenomenex, Torrance, CA, USA). The collected fractions were combined and lyophilized to retrieve the pure CoA analogs (**1**–**11**).

##### S-Acetonyl-coenzyme A (**1**)

A total of 47% yield. ^1^H-NMR (D_2_O, 400 MHz): δ 8.53 (s, 1H), 8.31 (s, 1H), 6.09 (d, J = 5.0 Hz, 1H), 4.77 (d, J = 5.5 Hz, 2H), 4.47 (s, 1H), 4.13 (s, 2H), 3.88 (s, 1H), 3.75–3.73 (m, 1H), 3.50–3.48 (m, 1H), 3.40 (s, 2H), 3.34 (t, J = 6.5 Hz, 2H), 3.21–3.16 (m, 2H), 2.48 (t, J = 6.5 Hz, 2H), 2.33 (t, J = 6.5 Hz, 2H), 2.15 (s, 3H), 0.20 (s, 3H), 0.68 (s, 3H); ^31^P-NMR (D_2_O, 162 MHz): δ 0.48, −11.06, −11.60. MALDI-TOF (+): calculated mass for C_24_H_41_N_7_O_17_P_3_S [M+H]^+^ 824.1487, found 824.4956.

##### Acetamide-coenzyme A (**2**)

A total of 67% yield. ^1^H-NMR (D_2_O, 400 MHz): δ 8.53 (s, 1H), 8.30 (s, 1H), 6.09 (d, J = 5.0 Hz, 1H), 4.87–4.77 (m, 2H), 4.47 (s, 1H), 4.13 (s, 2H), 3.88 (s, 1H), 3.72–3.74 (m, 1H), 3.43–3.53 (m, 1H), 3.34 (t, J = 6.5 Hz, 2H), 3.23 (t, J = 7.0 Hz, 2H), 3.14 (s, 2H), 2.57 (t, J = 6.5 Hz, 2H), 2.33 (t, J = 6.5 Hz, 2H), 0.80 (s, 3H), 0.68 (s, 3H); ^31^P-NMR (D_2_O, 162 MHz): δ 0.46, −11.06, −11.55; MALDI-TOF (−): calculated mass for C_23_H_38_N_8_O_17_P_3_S [M-H]^−^ 823.1294, found 823.4526.

##### 2-(S-Coenzyme A)-acetic Acid (**3**)

A total of 32% yield. ^1^H-NMR (D_2_O, 400 MHz): δ 8.55 (s, 1H), 8.31 (s, 1H), 6.09 (d, J = 5.5 Hz, 1H), 4.79–4.72 (m, 2H), 4.47 (s, 1H), 4.12 (s, 2H), 3.89 (s, 1H), 3.79–3.72 (m, 1H), 3.48–3.46 (m, 1H), 3.34 (t, J = 6.5 Hz, 2H), 3.05–3.03 (m, 4H), 2.59 (t, J = 6.5 Hz, 2H), 2.33 (t, J = 6.5 Hz, 2H), 0.80 (s, 3H), 0.67 (s, 3H); ^31^P-NMR (D_2_O, 162 MHz): δ −0.31, broad peak (−10.36)–(−11.99); MALDI-TOF (−): calculated mass for C_23_H_37_N_7_O_18_P_3_S [M-H]^−^ 824.1134, found 824.3917.

##### S-(Propanyl)-coenzyme A (**4**)

A total of 20% yield. ^1^H-NMR (D_2_O, 400 MHz): δ 8.53 (s, 1H), 8.31 (s, 1H), 6.09 (d, J = 5.0 Hz, 1H), 4.76–4.75 (m, 2H), 4.47 (s, 1H), 4.13 (s, 2H), 3.89 (s, 1H), 3.75–3.72 (m, 1H), 3.49–3.47 (m, 1H), 3.34 (t, J = 6.5 Hz, 2H), 3.21 (t, J = 6.5 Hz, 2H), 2.51 (t, J = 6.5 Hz, 2H), 2.38 (d, J = 7.5 Hz, 2H), 2.33 (t, J = 6.5 Hz, 2H), 1.41 (h, J = 7.5 Hz, 2H), 0.80 (s, 3H), 0.77 (t, J = 7.5 Hz, 3H), 0.68 (s, 3H); ^31^P-NMR (D_2_O, 162 MHz): δ 0.44, −11.04, 11.58; MALDI-TOF (−): calculated mass for C_24_H_41_N_7_O_16_P_3_S [M-H]^−^ 808.1549, found 808.3862.

##### S-(Benzyl)-coenzyme A (**5**)

A total of 32% yield. ^1^H-NMR (D_2_O, 400 MHz): δ 8.59 (s, 1H), 8.33 (s, 1H), 7.32–7.18 (m, 5H), 6.14 (d, J = 5.5 Hz, 1H), 4.84–4.79 (m, 2H), 4.55 (s, 1H), 4.22 (s, 2H), 4.97 (s, 1H), 3.83–3.81 (m, 1H), 3.66 (s, 2H), 3.58–3.56 (m, 1H), 3.40 (t, J = 6.5 Hz, 2H), 3.23 (t, J = 6.5 Hz, 2H), 2.49 (t, J = 6.5 Hz, 2H), 2.37 (t, J = 6.5 Hz, 2H), 0.88 (s, 3H), 0.75 (s, 3H); ^31^P-NMR (D_2_O, 162 MHz): δ 0.48, −11.06, −11.58; MALDI-TOF (−): calculated mass for C_28_H_41_N_7_O_16_P_3_S [M-H]^−^ 856.1549, found 856.4321.

##### S-(Propargyl)-coenzyme A (**6**)

A total of 33% yield. ^1^H-NMR (D_2_O, 400 MHz): δ 8.52 (s, 1H), 8.31 (s, 1H), 6.09 (d, J = 5.5 Hz, 1H), 4.77–4.71 (m, 2H), 4.47 (s, 1H), 4.14 (s, 2H), 3.89 (s, 1H), 3.75–3.73 (m, 1H), 3.50–3.48 (m, 1H), 3.34 (t, J = 6.5 Hz, 2H), 3.26 (t, J = 6.5 Hz, 2H), 3.18 (d, J = 2.5 Hz, 2H), 2.67 (t, J = 6.5 Hz, 2H), 2.49 (t, J = 2.5 Hz, 1H), 2.34 (t, J = 6.5 Hz, 2H), 0.81 (s, 3H), 0.69 (s, 3H); ^31^P-NMR (D_2_O, 162 MHz): δ 0.55, −11.12, −11.64; MALDI-TOF (−): calculated mass for C_24_H_37_N_7_O_16_P_3_S [M-H]^−^ 804.1236, found 804.3570.

##### S-(Allyl)-coenzyme A (**7**)

A total of 13% yield. ^1^H-NMR (D_2_O, 400 MHz): δ 8.55 (s, 1H), 8.31 (s, 1H), 6.09 (d, J = 5.5 Hz, 1H), 5.64 (ddt, J = 14.5, 9.5, 7.5 Hz, 2H), 5.02–4.97 (m, 2H), 4.76–4.67 (m, 2H), 4.47 (s, 1H), 4.13 (s, 2H), 3.89 (s, 1H), 3.74–3.72 (m, 1H), 3.48–3.46 (m, 1H), 3.34 (t, J = 6.5 Hz, 2H), 3.19 (t, J = 6.5 Hz, 2H), 3.02 (d, J = 7.5 Hz, 2H), 2.46 (t, J = 6.5 Hz, 2H), 2.33 (t, J = 6.5 Hz, 2H), 0.80 (s, 3H), 0.68 (s, 3H); ^31^P-NMR (D_2_O, 162 MHz): δ 0.34, −10.97, −10.55; MALDI-TOF (−): calculated mass for C_24_H_39_N_7_O_16_P_3_S [M-H]^−^ 806.1392, found 806.3840.

##### S-(Chloroacetonyl)-coenzyme A (**8**)

A total of 36% yield. ^1^H-NMR (D_2_O, 400 MHz): δ 8.64 (s, 1H), 8.40 (s, 1H), 6.18 (d, J = 5.5 Hz, 1H), 4.85–4.74 (m, 2H), 4.56 (s, 1H), 4.22 (s, 2H), 3.98 (s, 1H), 3.82–3.80 (m, 1H), 3.58–3.56 (m, 3H), 3.43 (t, J = 6.5 Hz, 2H), 3.30 (t, J = 6.5 Hz, 2H), 2.66 (s, 2H), 2.60 (t, J = 6.5 Hz, 2H), 2.43 (t, J = 6.5 Hz, 2H), 0.89 (s, 3H), 0.77 (s, 3H); MALDI-TOF (−): calculated mass for C_24_H_38_ClN_7_O_17_P_3_S [M-H]^−^ 856.0952, found 856.3394.

##### S-(Ethylamino)-coenzyme A (**9**)

A total of 63% yield. ^1^H NMR (500 MHz, D_2_O) δ 8.55 (s, 1H), 8.31 (s, 1H), 6.10 (d, J = 5.5 Hz, 1H), 4.77–4.71 (m, 2H), 4.48 (s, 1H), 4.13 (s, 2H), 3.92 (s, 1H), 3.73 (dd, J = 9.5, 4.5 Hz, 1H), 3.47 (dd, J = 9.5, 4.5 Hz, 1H), 3.41–3.32 (m, 2H), 3.25 (td, J = 6.5, 1.5 Hz, 2H), 3.08 (t, J = 6.5 Hz, 2H), 2.73 (t, J = 6.5 Hz, 2H), 2.57 (t, J = 6.5 Hz, 2H), 2.35 (t, J = 6.5 Hz, 2H), 0.82 (s, 3H), 0.71 (s, 3H). ^31^P NMR (202 MHz, D_2_O) δ 0.37, −10.22, −10.75. ESI-HRMS (−) calculated mass for C_23_H_40_N_8_O_16_P_3_S [M-H]^−^ 809.1501, found 809.1485.

##### S-(4-Methoxyphenacetonyl)-coenzyme A (**10**)

Obtained 3.47 mg (3.79 μmol, 29%). ^1^H NMR (500 MHz, D_2_O) δ 8.49 (s, 1H), 8.24 (s, 1H), 7.80–7.72 (m, 2H), 6.89–6.81 (m, 2H), 6.02 (d, J = 5.5 Hz, 1H), 4.82–4.69 (m, 2H), 4.50–4.43 (m, 1H), 4.19–4.10 (m, 2H), 3.91 (s, 1H), 3.84 (s, 2H), 3.79–3.75 (m, 1H), 3.74 (s, 3H), 3.48 (dd, J = 9.5, 4.5 Hz, 1H), 3.30 (t, J = 6.5 Hz, 2H), 3.22 (t, J = 6.5 Hz, 2H), 2.55 (t, J = 6.5 Hz, 2H), 2.28 (t, J = 6.5 Hz, 2H), 0.82 (s, 3H), 0.68 (s, 3H). ^31^P NMR (202 MHz, D_2_O) δ 0.18, −10.33, −10.91. ESI-HRMS (−) calculated mass for C_30_H_43_N_7_O_18_P_3_S [M-H]^−^ 914.1604, found 914.1584.

##### S-(Phenacetonyl)-coenzyme A (**11**)

Obtained 3.14 mg (3.54 μmol, 27%). ^1^H NMR (500 MHz, D_2_O) δ 8.50 (s, 1H), 8.25 (s, 1H), 7.80–7.77 (m, 2H), 7.51 (t, J = 7.5 Hz, 1H), 7.41–7.31 (m, 2H), 6.04 (d, J = 5.5 Hz, 1H), 4.81–4.71 (m, 2H), 4.70 (td, J = 0.5, 0.5 Hz, 1H), 4.47 (q, J = 2.5 Hz, 1H), 4.19–4.11 (m, 2H), 3.90 (d, J = 2.0 Hz, 3H), 3.75 (dd, J = 9.5, 5.0 Hz, 1H), 3.49 (dd, J = 9.5, 4.5 Hz, 1H), 3.30 (t, J = 6.5 Hz, 2H), 3.22 (t, J = 6.5 Hz, 2H), 2.54 (t, J = 6.5 Hz, 2H), 2.28 (t, J = 6.5 Hz, 2H), 0.81 (s, 3H), 0.68 (s, 3H). ^31^P NMR (202 MHz, D_2_O) δ 0.17, −10.40, −10.88. ESI-HRMS (−) calculated mass for C_29_H_41_N_7_O_17_P_3_S [M] 884.1498, found 884.1458.

### 4.3. KAT Enzymes Expression and Purification

Recombinant KAT8 and GCN5 were expressed and purified as we previously described [79]. The expression and purification of HAT1 were carried out following a previously reported procedure [71]. Briefly, N-terminal His-tagged human HAT1 (amino acids 22-341) was transformed into *E. coli* Rosetta BL21 (DE3)pLysS cells, and the expression was induced by addition of 1.0 mM 3-isopropyl-1-thio-D-galactopyranoside (IPTG) and cultured overnight at 15 °C. Harvested cells were resuspended in 50 mM sodium phosphate buffer pH 7.8, 250 mM NaCl, 5 mM imidazole, 5% glycerol, and 5 mM β-mercaptoethanol and subsequently lysed by sonication. The cell lysate was then incubated with Ni-NTA beads, and the beads were washed with buffer (20 mM Tris-HCl pH 7.8, 250 mM NaCl, 20 mM imidazole, and 5% glycerol). The protein was eluted with 20 mM Tris-HCl (pH 7.8, 250 mM NaCl, 250 mM imidazole, and 5% glycerol). The eluted protein was concentrated using a spinfilter device (Cytiva, Uppsala, Sweden, MWCO 10 kDa) and further purified by gel filtration chromatography using a Superdex 75 column (Cytiva, Uppsala, Sweden) and running buffer (20 mM Tris pH 7.5, 250 mM NaCl and 10 mM β-mercaptoethanol) at 0.5 mL min−1 flow speed. The purity of the eluted protein was assessed with SDS-PAGE. Pure fractions were pooled, rapidly flash-frozen, and stored at −80 °C.

### 4.4. Histone Peptide Substrate Synthesis and Purification

The 13-mer H3K9 peptide (H3 1–13 residues, sequence ARTKQTARKSTGG), 15-mer H4K16 peptide (H4 13–27 residues; sequence GGAKRHRKVLRDNIQ) and 20-mer H4K12 peptide (H4 1–20 residues; sequence SGRGKGGKGLGKGGAKRHRK) were chain-assembled on Rink amide resin (0.78 mmol g^−1^ loading capacity) by Fmoc-based solid-phase peptide synthesis (SPPS) chemistry, using Gyros PurePep® Chorus Peptide Synthesiser (Tucson, AZ, USA) on a 0.05 mmol scale. Couplings were carried out by adding a mixture of the Fmoc-protected amino acid (3.0 equiv.), HATU (3.0 equiv.), and DIPEA (6.0 equiv.) in DMF for 10 min at 75 °C. Fmoc-deprotection was achieved by a solution of 20 % (*v*/*v*) piperidine in DMF for 4 min at 50 °C. Following the final Fmoc-deprotection step, the resin was washed with DCM (3×) and methanol (3×) and dried over diethyl ether. Cleavage of the peptide from the resin was then carried out with a mixture of 95 % TFA, 2.5 % TIPS, and 2.5 % MQ for 4 h. The crude peptide was precipitated by addition of cold diethyl ether, and after suspension, it was centrifuged for 5 min at 5000 rpm in an Eppendorf 5804R centrifuge (Eppendorf, Hamburg, Germany), after which the supernatant was removed. Washing of the remaining solid with cold Et2O and centrifugation were repeated twice more. The crude peptide was dissolved in a mixture of ACN in H_2_O and purified using preparative reverse-phase HPLC (RP-HPLC) using a gradient of H_2_O + 0.1% TFA and ACN + 0.1% TFA from 5% ACN to 30% ACN over 30 min at 4 mL/min using a Gemini 10 μm NX-C18 110Å LC column (Phenomenex, Torrance, CA, USA). Analytical RP-HPLC was carried out on a Gemini 5 μm C18 110Å LC column (Phenomenex) at a flow rate of 1 mL/min. Analytical injections were monitored at 215 nm. The mass of the peptide was confirmed by mixing with a 1:2 with α-Cyano-4-hydroxycinnamic acid (α-CHCA) matrix and loaded onto an MTP 384 polished steel target to be analyzed by MALDI-TOF.

### 4.5. MALDI-TOF Inhibition Assays

For GCN5 and KAT8: The acetylation of the H3K9 peptide (for GCN5) and the H4K16 peptide (for KAT8) was performed as follows: First, the enzyme (500 nM final concentration) and the CoASH analogs at a range of final concentrations of 100 nM–5 mM (from stocks in MilliQ water stored at −80 °C; note: compounds remained stable for >1 year) were preincubated for 10 min in reaction buffer (50 mM HEPES, 0.1 mM EDTA, pH 8.0). After preincubation, histone peptide (final concentration 10 µM) and AcCoA (final concentration 40 µM) were added, at a final volume of 20 µL. The mixture was incubated for 30 min at 37 °C, after which it was quenched by the addition of 20 µL of 10% TFA in MilliQ-water. For MALDI-TOF MS analysis, a 2 µL aliquot of the quenched reaction mixture was mixed with 2 µL of α-CHCA matrix, and from this, 2 µL was loaded onto an MTP 384 polished steel target to be analyzed by MALDI-TOF. The total peak area of unacetylated and acetylated state, with all isotopes and adducts, was used for determination of enzymatic activity in the presence of each inhibitor relative to the activity in their absence. The assay was performed in duplicates, and each point is presented as the mean of the duplicates, plus the standard deviation.

For HAT1: The acetylation of the H4K12 peptide was performed as follows: First, the enzyme (200 nM final concentration) and the CoASH analogs at a range of final concentrations of 1 nM–100 μM mM (from 1 mM stocks in MilliQ water) were preincubated for 10 min in reaction buffer (50 mM HEPES, 0.1 mM EDTA, 1 mM DTT, pH 8.0). After preincubation, histone peptide (final concentration 10 µM) and AcCoA (final concentration 20 µM) were added, at a final volume of 20 µL. The mixture was incubated for 20 min at 37 °C, after which it was quenched by the addition of 20 µL of 10% TFA in MilliQ-water. For MALDI-TOF MS analysis, a 2 µL aliquot of the quenched reaction mixture was mixed with 2 µL of α-CHCA matrix, and from this, 2 µL was loaded onto an MTP 384 polished steel target to be analyzed by MALDI-TOF. The total peak area of unacetylated and acetylated state, with all isotopes and adducts, was used for determination of enzymatic activity in the presence of each inhibitor relative to the activity in their absence. The assay was performed in duplicates, and each point is presented as the mean of the duplicates, plus the standard error.

### 4.6. Molecular Docking

The enzymes used for molecular docking studies were imported into the Maestro module of the Schrödinger suite (version 2019-1) (PDB: 1Z4R for GCN5, PDB: 2GIV for KAT8, and PDB: 2P0W for HAT1) [80]. The Protein Preparation Module was used to add missing side-chain atoms, add hydrogen atoms, and determine protonation states of the enzymes. The enzyme and AcCoA ligand were preprocessed by a restrained minimization using the OPLS2005 force field before and after docking. AcCoA was replaced by the ketone-substituted CoASH (inhibitor **1**). AcCoA and the ketone-substituted variant were docked to all three enzymes using Glide (version 2023-3) [81].

## 5. Conclusions

A panel of diverse S-functionalized CoASH analogs was synthesized in one step and tested against three biomedically important epigenetic enzymes, histone acetyltransferases GCN5, KAT8, and HAT1. These compounds were compared to CoASH and, in the case of GCN5 and KAT8, most of them exhibited a much higher potency than the reference CoASH compound. Notably, the two most potent compounds against the three enzymes were ketone-substituted **1** and acetamide-substituted **2**, both showing higher potency with GCN5, KAT8, and HAT1 and displaying comparable activity to previously published CoASH-based inhibitors of KATs. Additionally, phenylacetyl-substituted **10** and **11** were both generally more active than the carbonyl-free benzyl-substituted **5**. This apparent difference in potency between the carbonylated and carbonyl-free compounds points towards the importance of the carbonyl group and its interaction with surrounding residues on the affinity of AcCoA and its analogs within the AcCoA binding site of KATs. In the case of KAT8 and GCN5, it also reinforces the possible role of the conserved cysteine not only in catalyzing lysine acetylation but in binding of the cosubstrate. Furthermore, some functionalized-CoASH structures proved to be significantly more selective towards one KAT. These results point to a potential for fine-tuning the structure of CoASH derivatives, providing ample space for further functionalization that might lead to improved potency and selectivity. More research into synthesizing CoASH analogs with **1** and **2** as lead compounds could pave the way toward inhibitors with improved selectivity and potency at the nM scale, without addressing the complexity of peptidomimetic structures of substrate-competitive inhibitors. Overall, our results offer a useful contribution to development of chemical probes with high potency towards the biomedically important KAT enzymes.

## Figures and Tables

**Table 1:** IC_50_ values for CoASH and its analogs against GCN5, KAT8, and HAT1.

Compound	IC_50_ (μΜ)		
	GCN5	KAT8	HAT1
CoASH	581	153	7.3
**1**	10.9	13.6	5.8
**2**	14.1	32.2	3.9
**3**	>100	>100	>100
**4**	>100	37.4	15.7
**5**	>100	>100	>100
**6**	85.0	>100	>100
**7**	>100	>100	>100
**8**	14.1	70.5	16.6
**9**	>100	50.8	>100
**10**	60.4	40.1	>100
**11**	27.7	63.9	>100

## Data Availability

The data presented in this study are available in Supplementary Materials.

## Supplementary Materials

The following supporting information can be downloaded at: https://www.mdpi.com/article/10.3390/molecules31030477/s1, Figure S1: Dose-response curves for inhibition of GCN5 by (a) **1**, (b) **2**, (c) **6**, (d) **8**, (e) **10**, (f) **11**, in comparison to CoASH; Figure S2: Dose-response curves for inhibition of KAT8 by (a) **1**, (b) **2**, (c) **4**, (d) **8**, (e) **9**,(f) **10**, (g) **11**, in comparison to CoASH; Figure S3: Dose-response curves for inhibition of HAT1 by (a) **1**, (b) **2**, (c) **4**, (d) **8**, in comparison to CoASH; Figure S4: (a) Superimposed structures of the docked inhibitor **1** (blue) in complex with KAT8 (grey) and the X-ray structure of propionyl-CoA (orange) in complex with KAT8 (grey, PDB: 5WCI). (b) Superimposed structures of the docked inhibitor **1** (blue) in complex with GCN5 (purple) and the X-ray structure of propionyl-CoA (orange) in complex with GCN5 (purple, PDB: 5H84); Figure S5: ^1^H-NMR of **1**; Figure S6: ^31^P-NMR of **1**; Figure S7: ^1^H-NMR of **2**; Figure S8: ^31^P-NMR of **2**; Figure S9: ^1^H-NMR of **3**; Figure S10: ^31^P-NMR of **3**; Figure S11: ^1^H-NMR of **4**; Figure S12: ^31^P-NMR of **4**; Figure S13: ^1^H-NMR of **5**; Figure S14: ^31^P-NMR of **5**; Figure S15: ^1^H-NMR of **6**; Figure S16: ^31^P-NMR of **6**; Figure S17: ^1^H-NMR of **7**; Figure S18: ^31^P-NMR of **7**; Figure S19: ^1^H-NMR of **8**; Figure S20: ^1^H-NMR of **9**; Figure S21: ^31^P-NMR of **9**; Figure S22: ^1^H-NMR of **10**; Figure S23: ^31^P-NMR of **10**; Figure S24: ^1^H-NMR of **11**; Figure S25: ^31^P-NMR of **11**; Figure S26: Analytical HPLC spectrum of **1**; Figure S27: Analytical HPLC spectrum of **2**; Figure S28: Analytical HPLC spectrum of **3**; Figure S29: Analytical HPLC spectrum of **4**; Figure S30: Analytical HPLC spectrum of **5**; Figure S31: Analytical HPLC spectrum of **6**; Figure S32: Analytical HPLC spectrum of **7**; Figure S33: Analytical HPLC spectrum of **8**; Figure S34: Analytical HPLC spectrum of **9**; Figure S35: Analytical HPLC spectrum of **10**; Figure S36: Analytical HPLC spectrum of **11**.
