# Supplementary material for: Inhibition of Histone Lysine Acetyltransferases by Coenzyme A Analogs
Source: Molecules. 2026 Jan 29;31(3):477. doi: 10.3390/molecules31030477 (PMC12899889; doi:10.3390/molecules31030477)
Supplement: Supplementary file 1 [file molecules-31-00477-s001.zip › molecules-4081957-supplementary.pdf]

Supporting Information

1. Additional dose-response curves

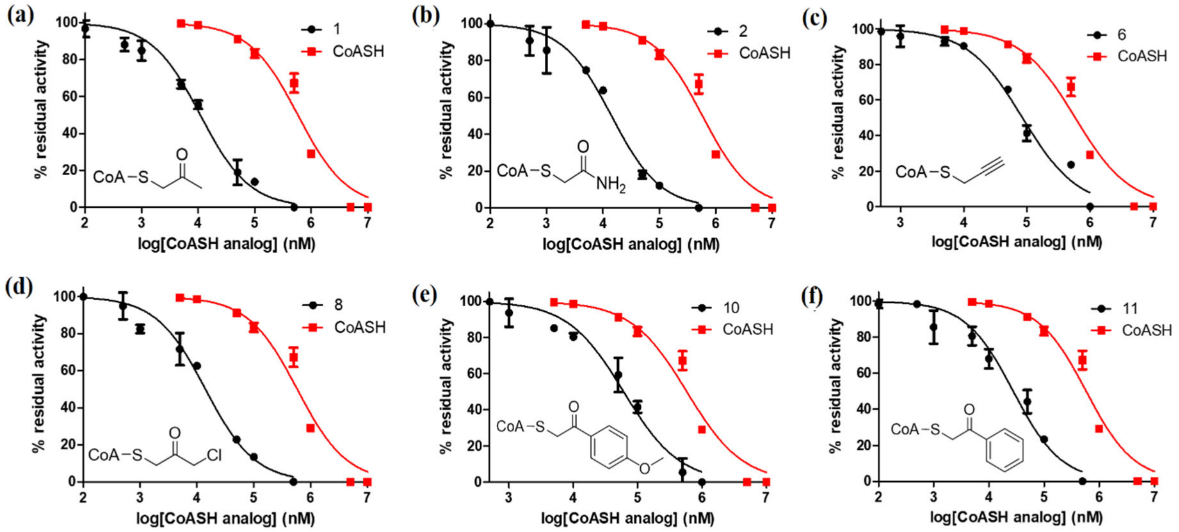

Figure S1. Dose-response curves for inhibition of GCN5 by (a) **1**, (b) **2**, (c) **6**, (d) **8**, (e) **10**, (f) **11**, in comparison to CoASH.

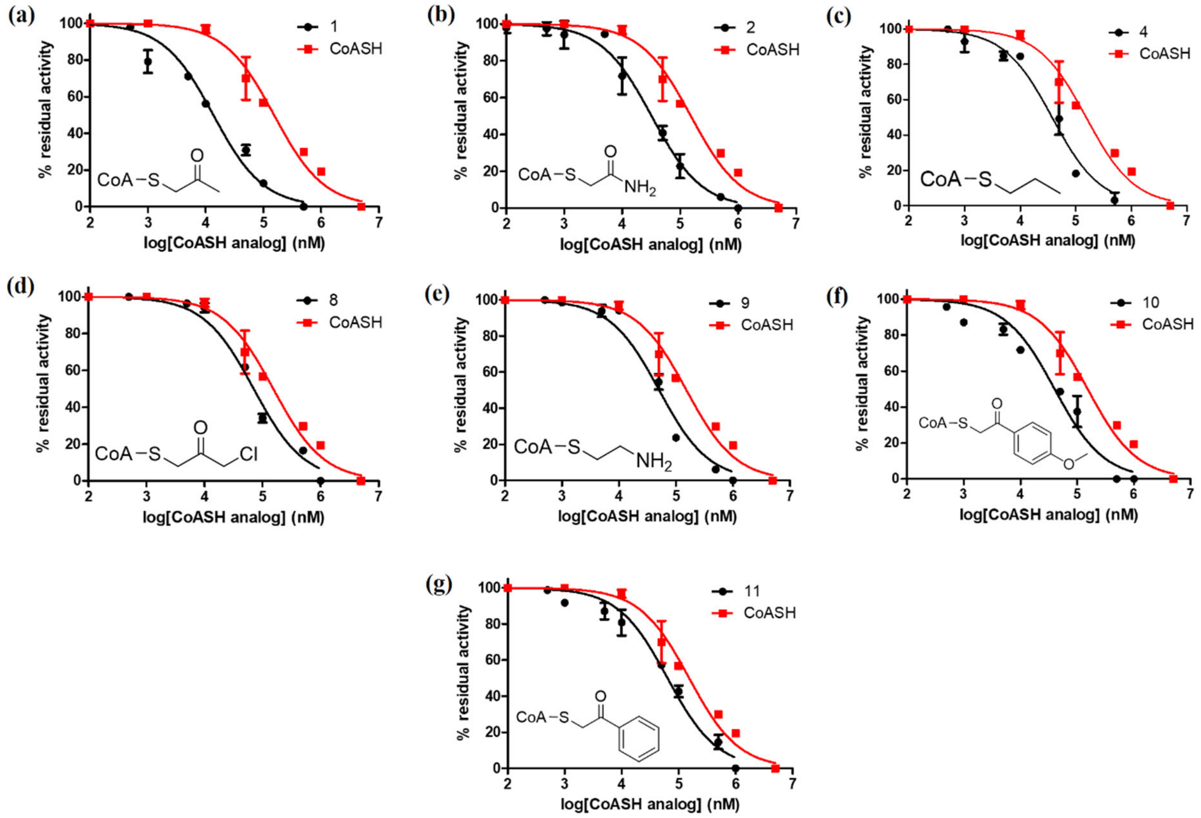

Figure S2. Dose-response curves for inhibition of KAT8 by (a) **1**, (b) **2**, (c) **4**, (d) **8**, (e) **9**,(f) **10**, (g) **11**, in comparison to CoASH.

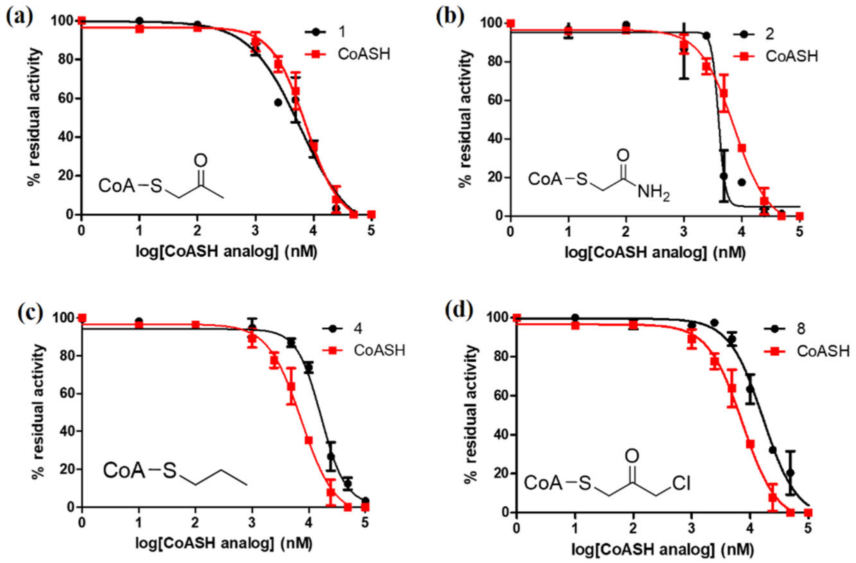

Figure S3. Dose-response curves for inhibition of HAT1 by (a) **1**, (b) **2**, (c) **4**, (d) **8**, in comparison to CoASH.

2. Molecular docking

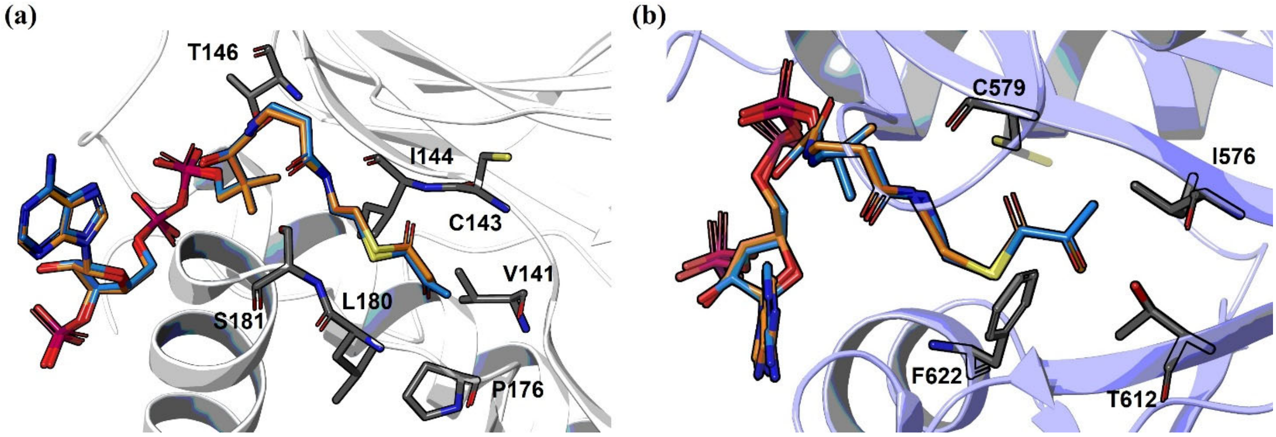

Figure S4. (a) Superimposed structures of the docked inhibitor **1** (blue) in complex with KAT8 (grey) and the X-ray structure of propionyl-CoA (orange) in complex with KAT8 (grey, PDB: 5WCI). (b) Superimposed structures of the docked inhibitor **1** (blue) in complex with GCN5 (purple) and the X-ray structure of propionyl-CoA (orange) in complex with GCN5 (purple, PDB: 5H84).

3. Characterization data of synthesized compounds

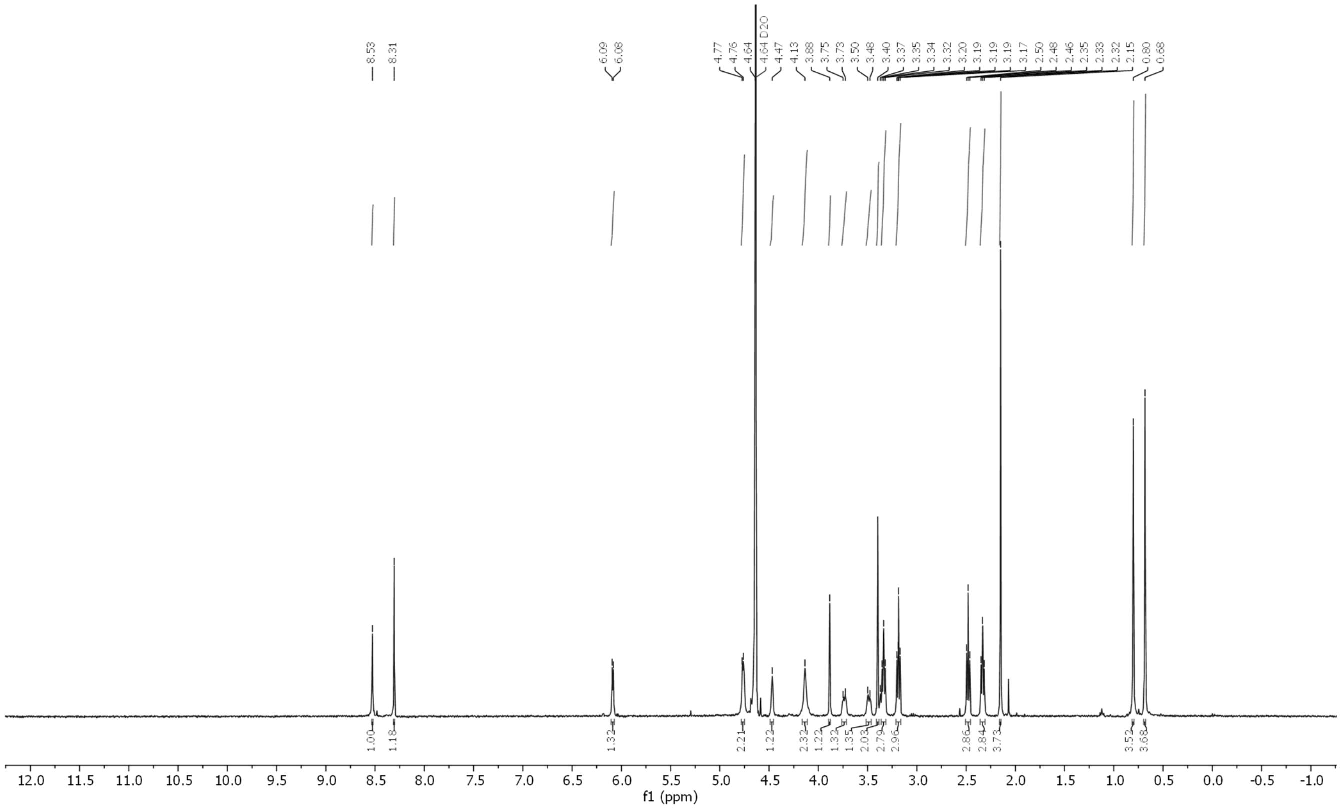

Figure S5. ^{1}H-NMR of **1**.

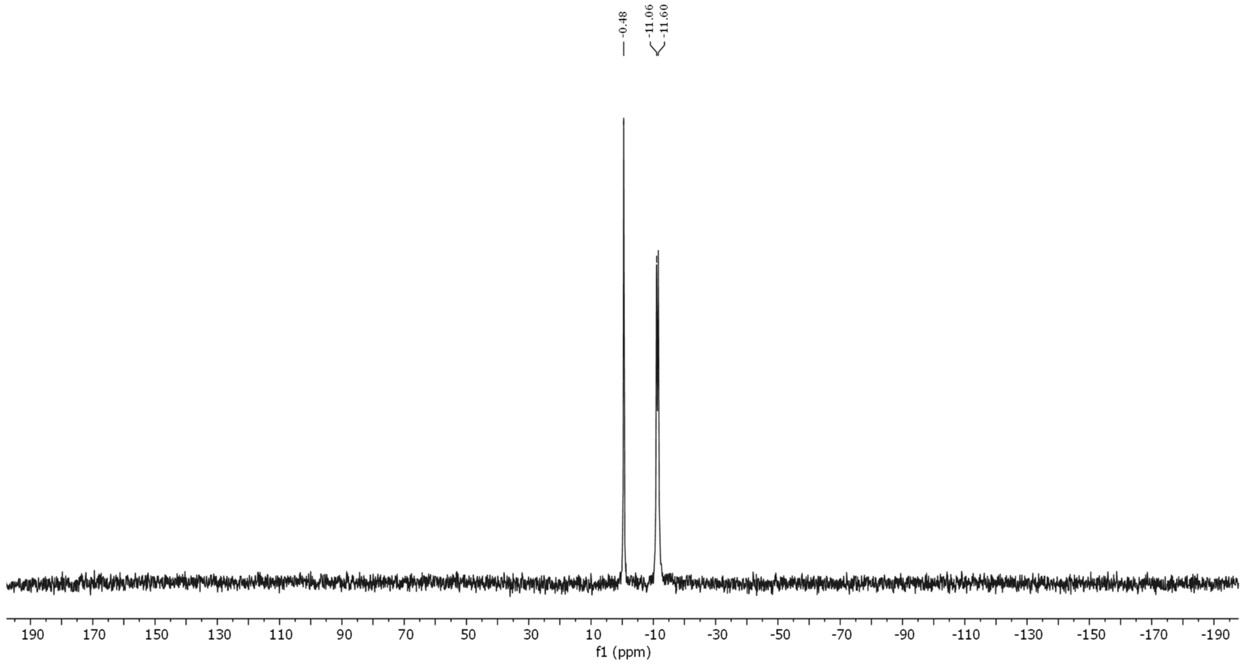

Figure S6. ^{31}P-NMR of **1**.

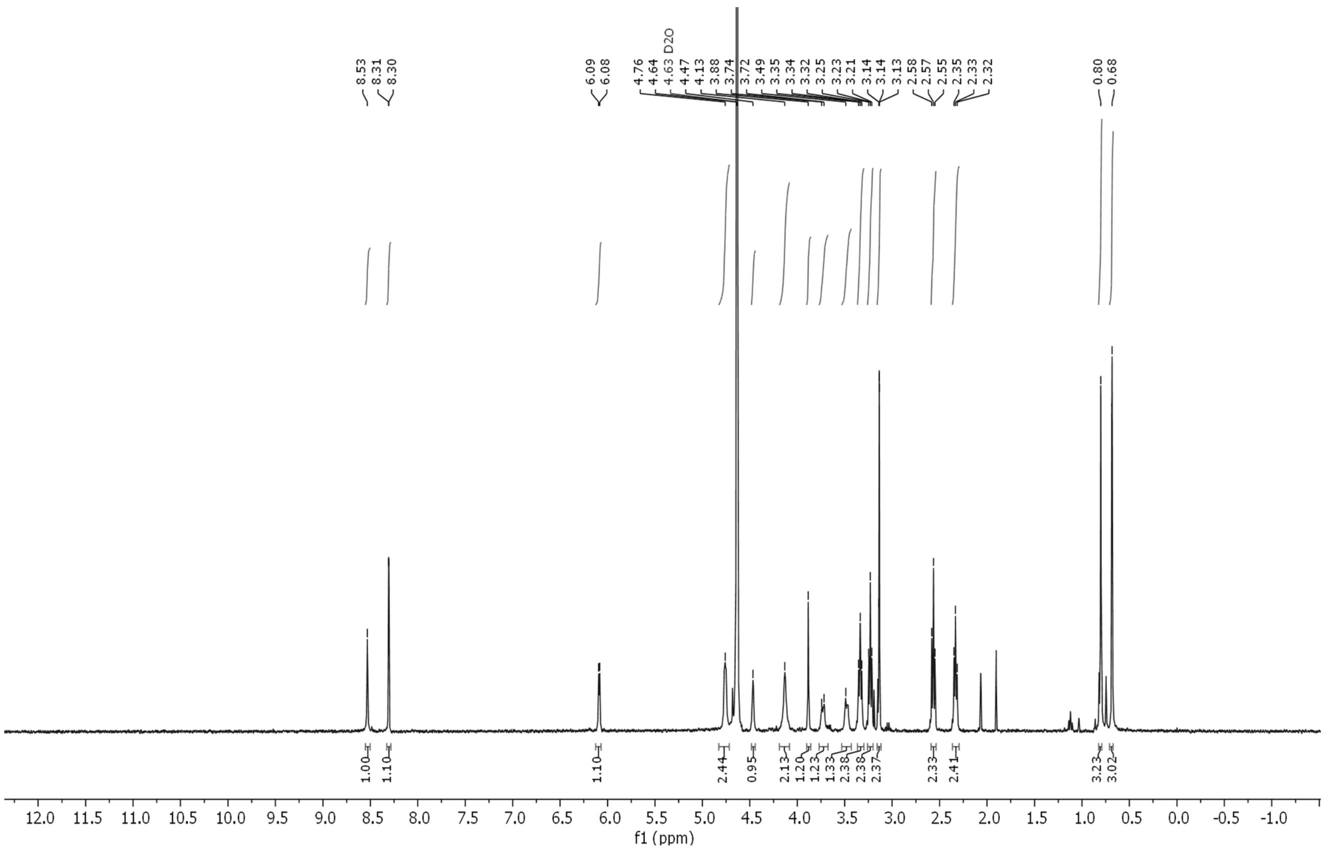

Figure S7. ^{1}H-NMR of **2**.

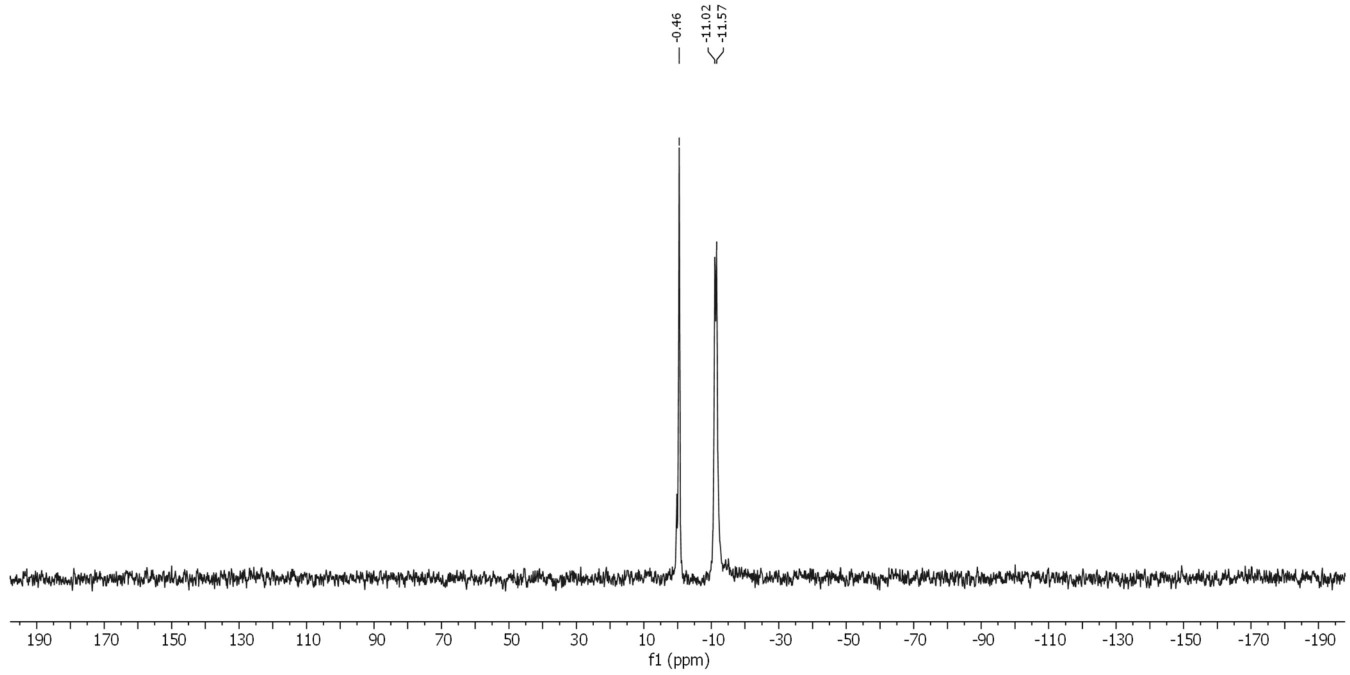

Figure S8. ^{31}P-NMR of **2**.

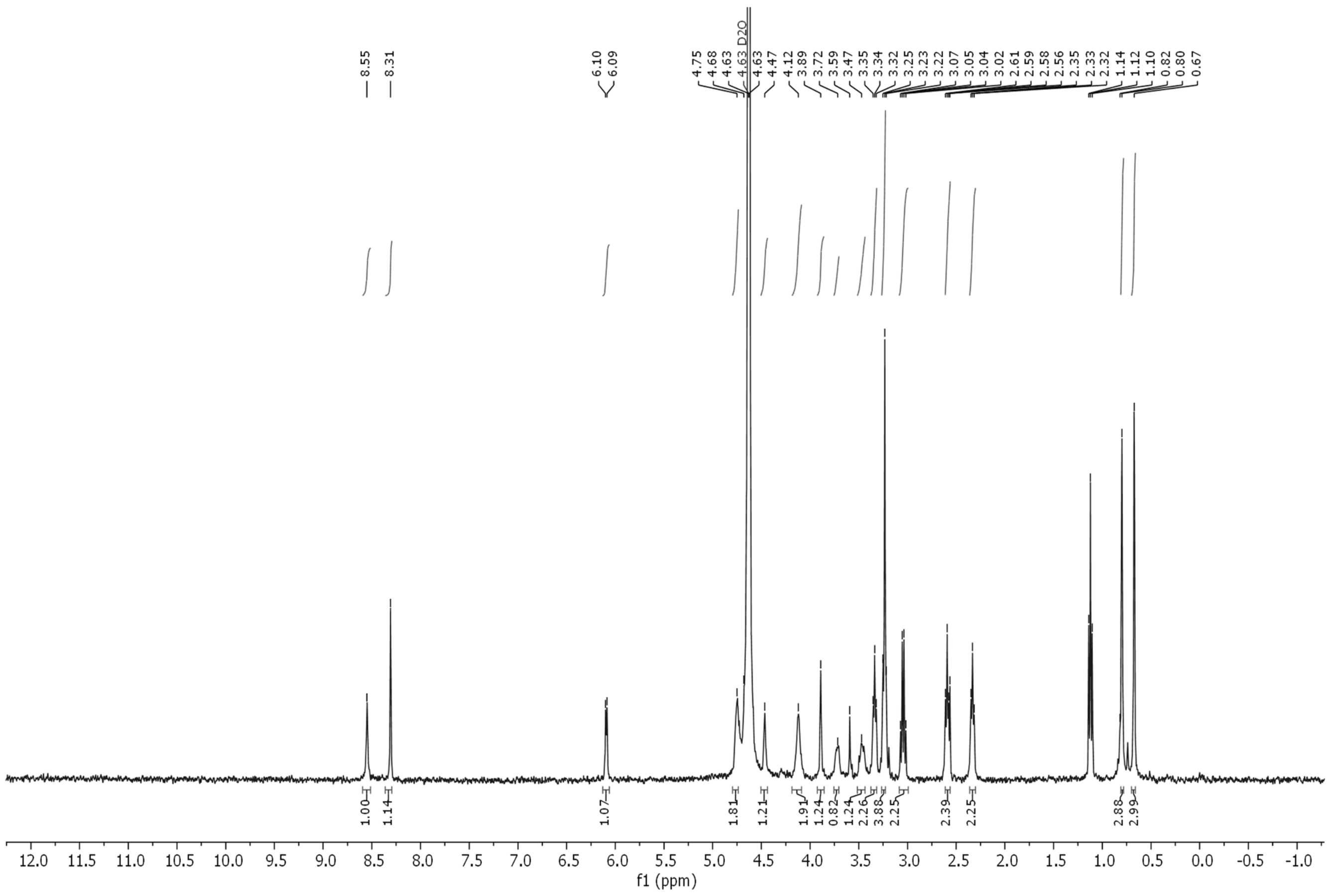

Figure S9. ^{1}H-NMR of **3**.

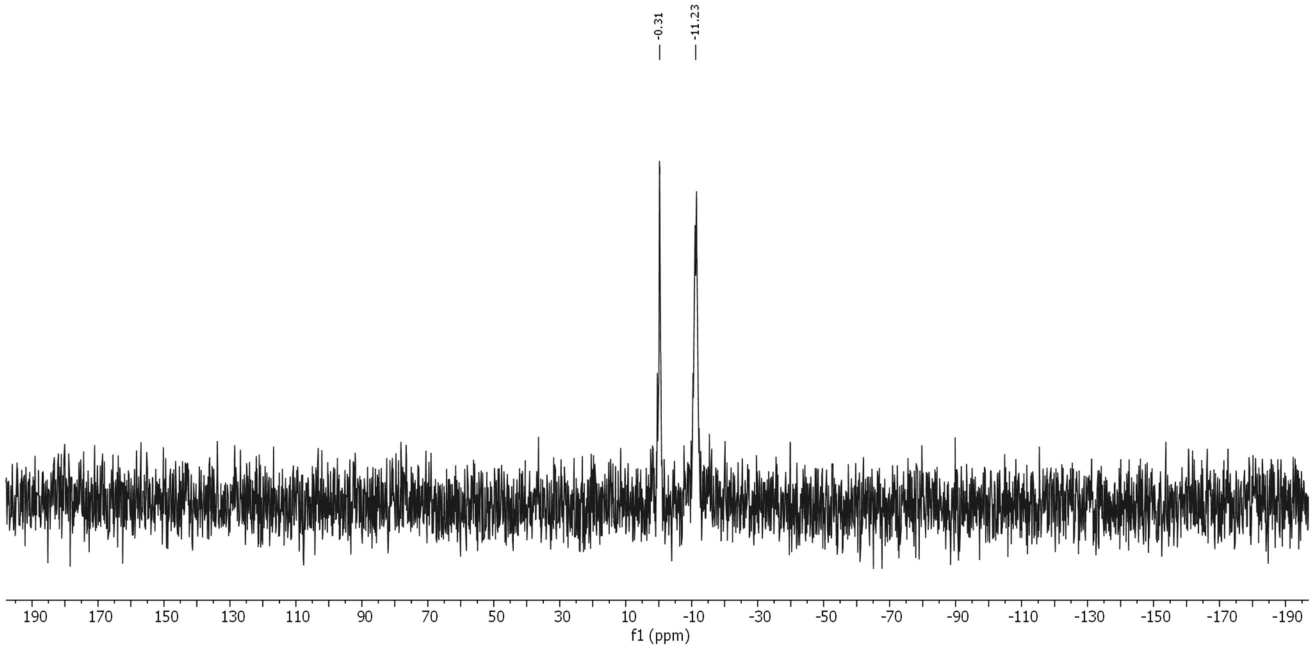

Figure S10. ^{31}P-NMR of **3**.

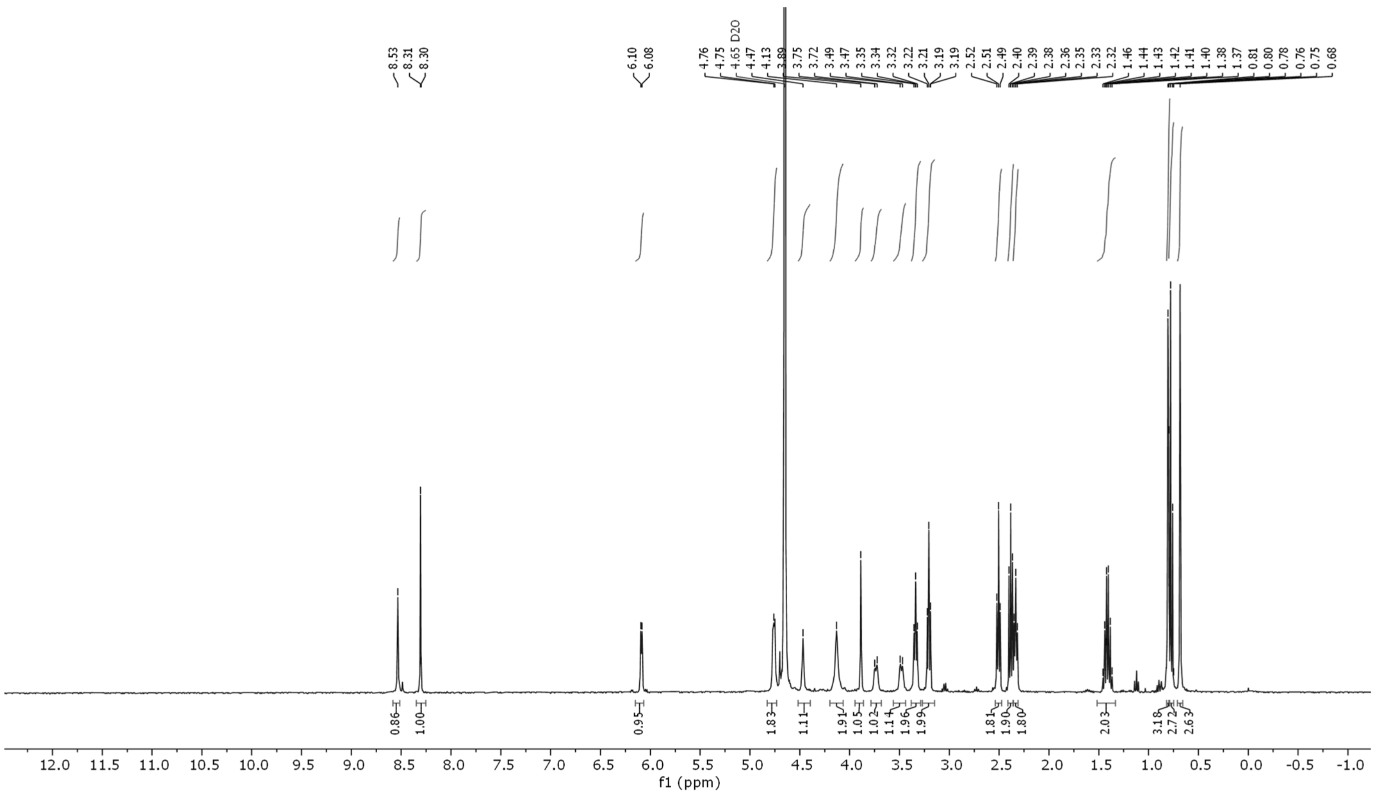

Figure S11. ^{1}H-NMR of **4**.

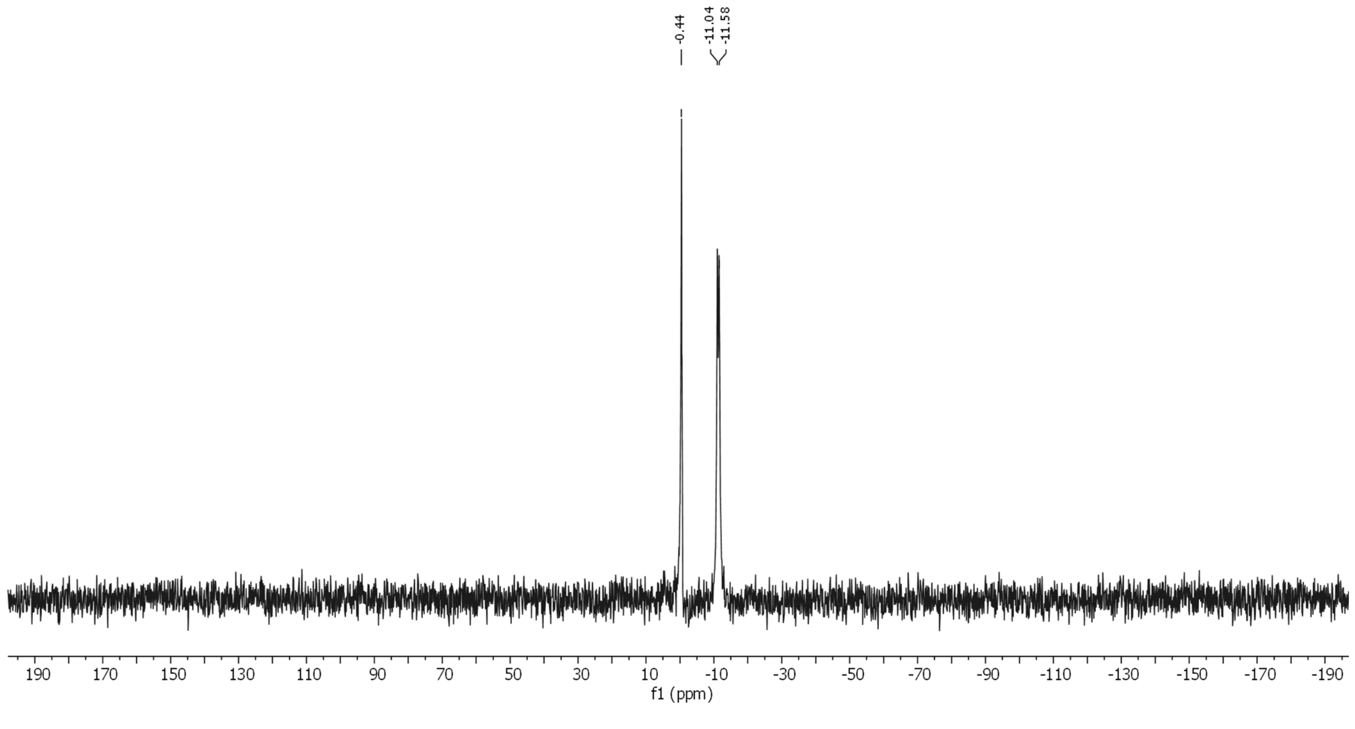

Figure S12. ^{31}P-NMR of **4**.

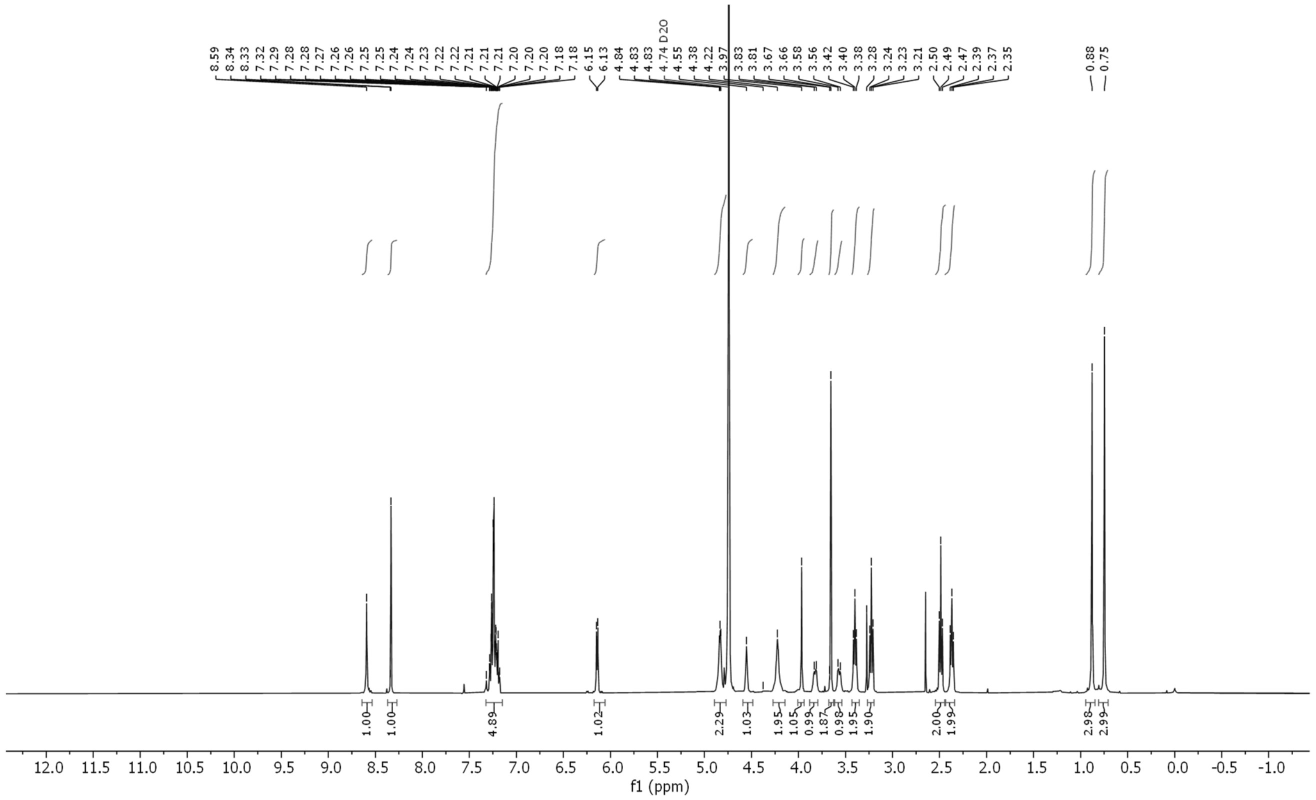

Figure S13. ^{1}H-NMR of **5**.

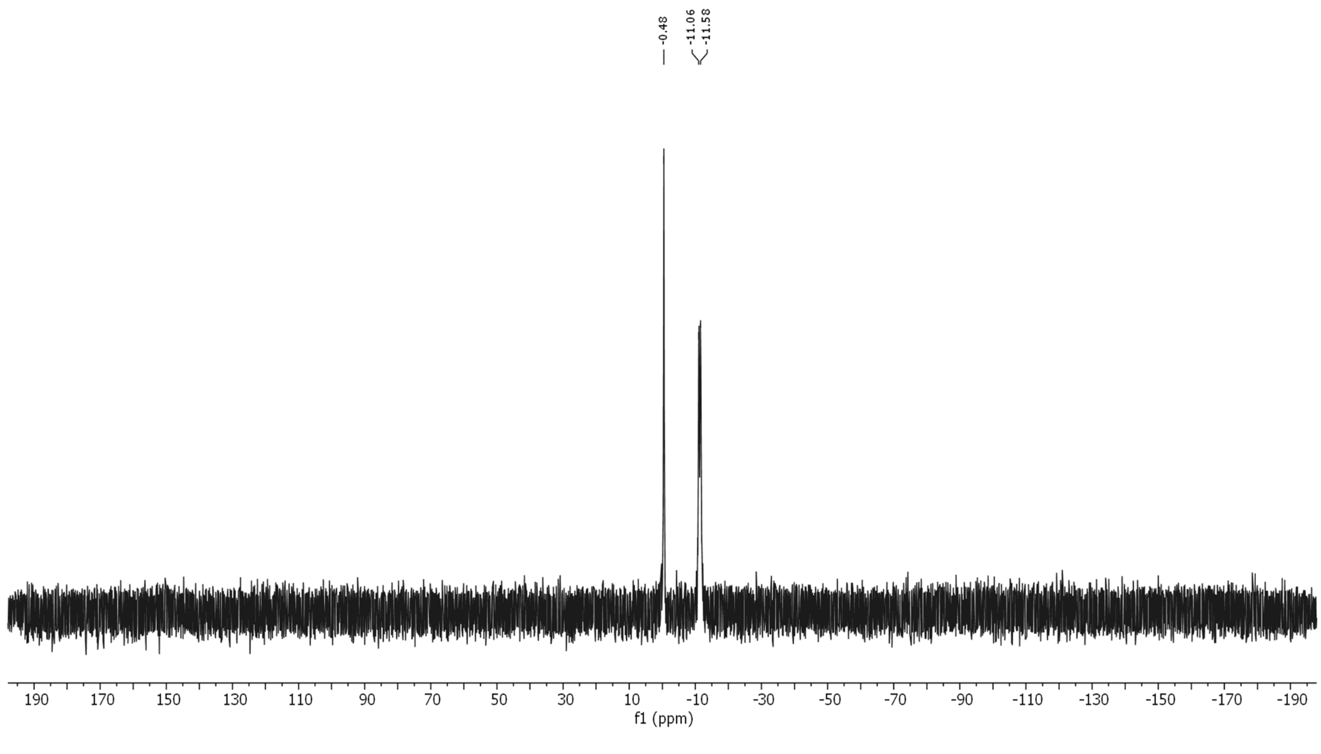

Figure S14. ^{31}P-NMR of **5**.

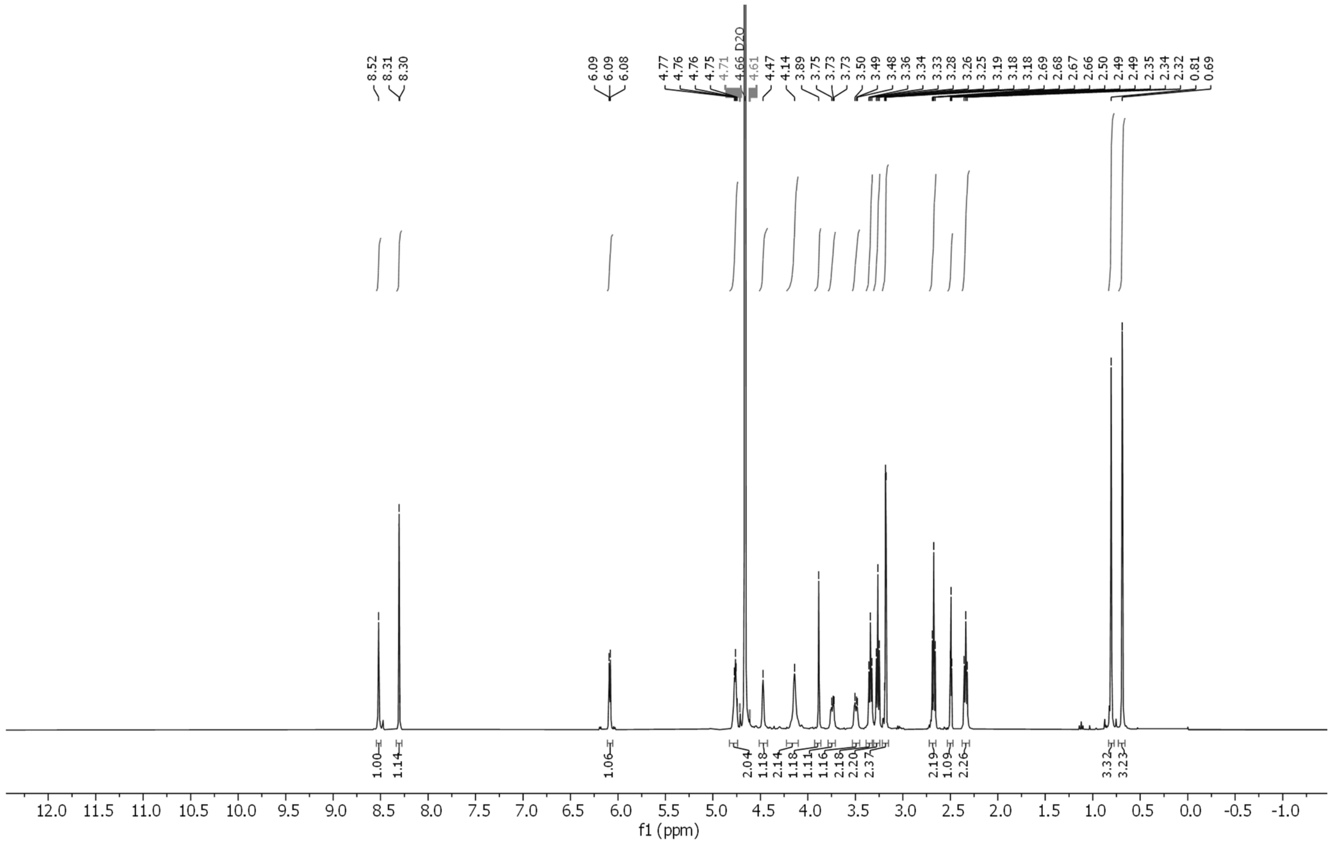

Figure S15. ^{1}H-NMR of **6**.

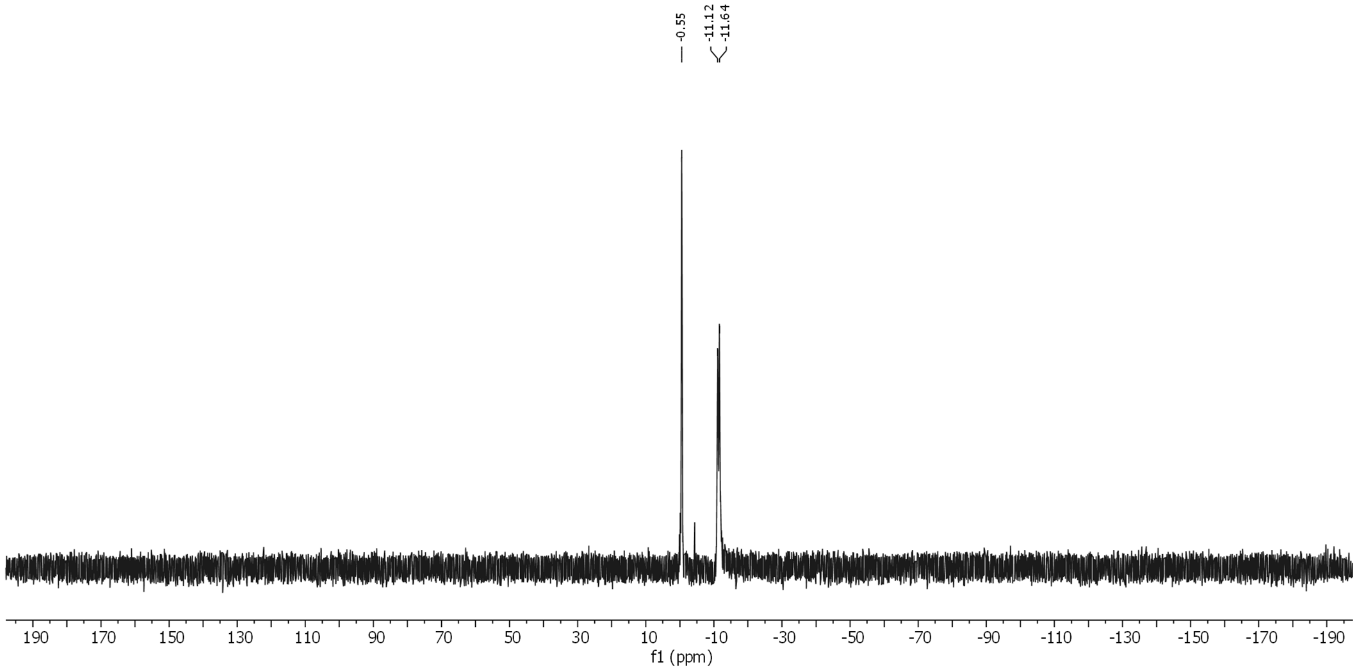

Figure S16. ^{31}P-NMR of **6**.

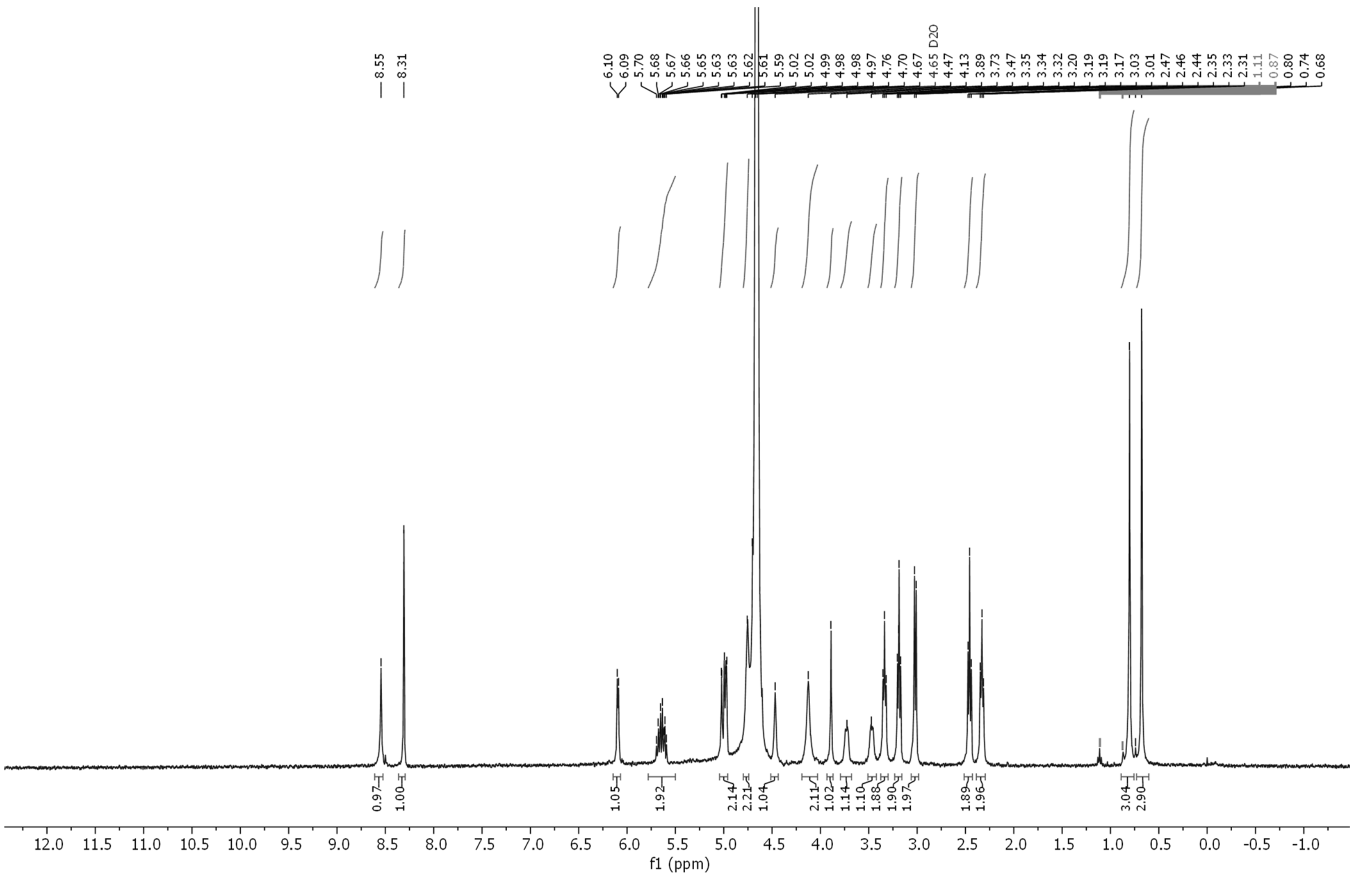

Figure S17. ^{1}H-NMR of **7**.

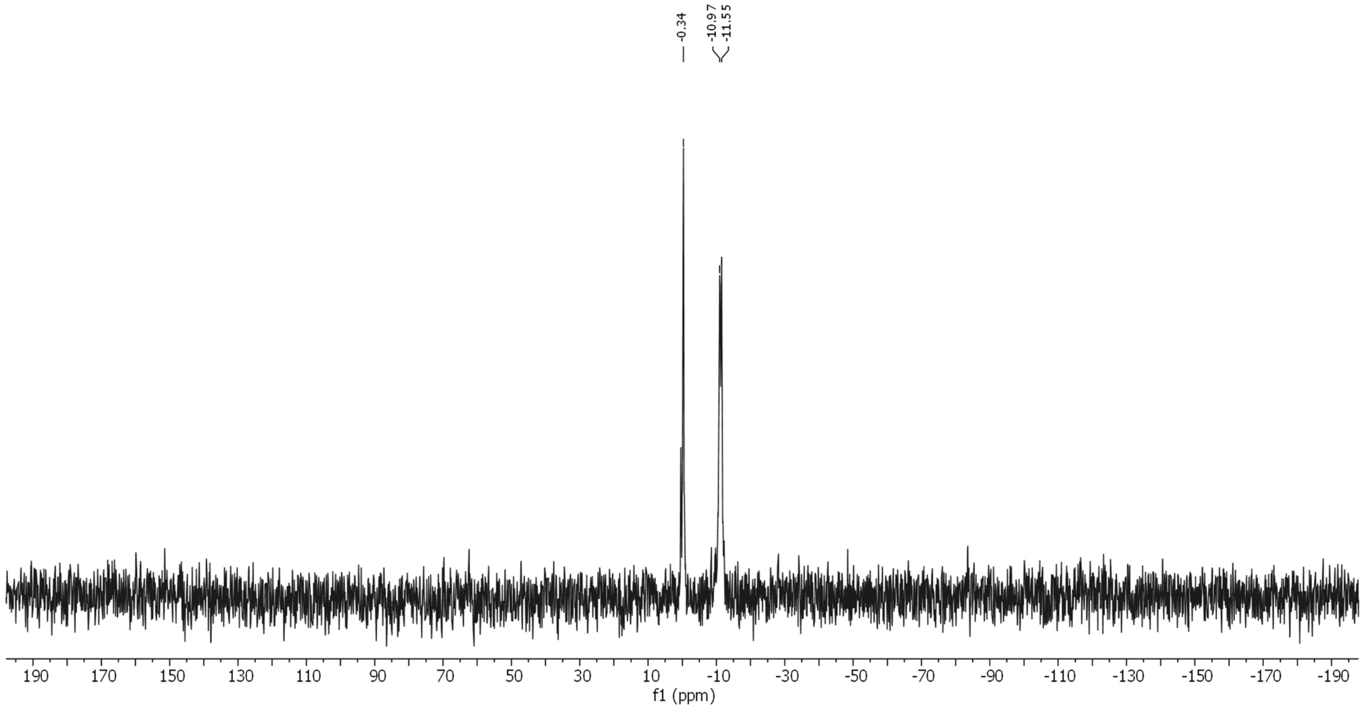

Figure S18. ^{31}P-NMR of **7**.

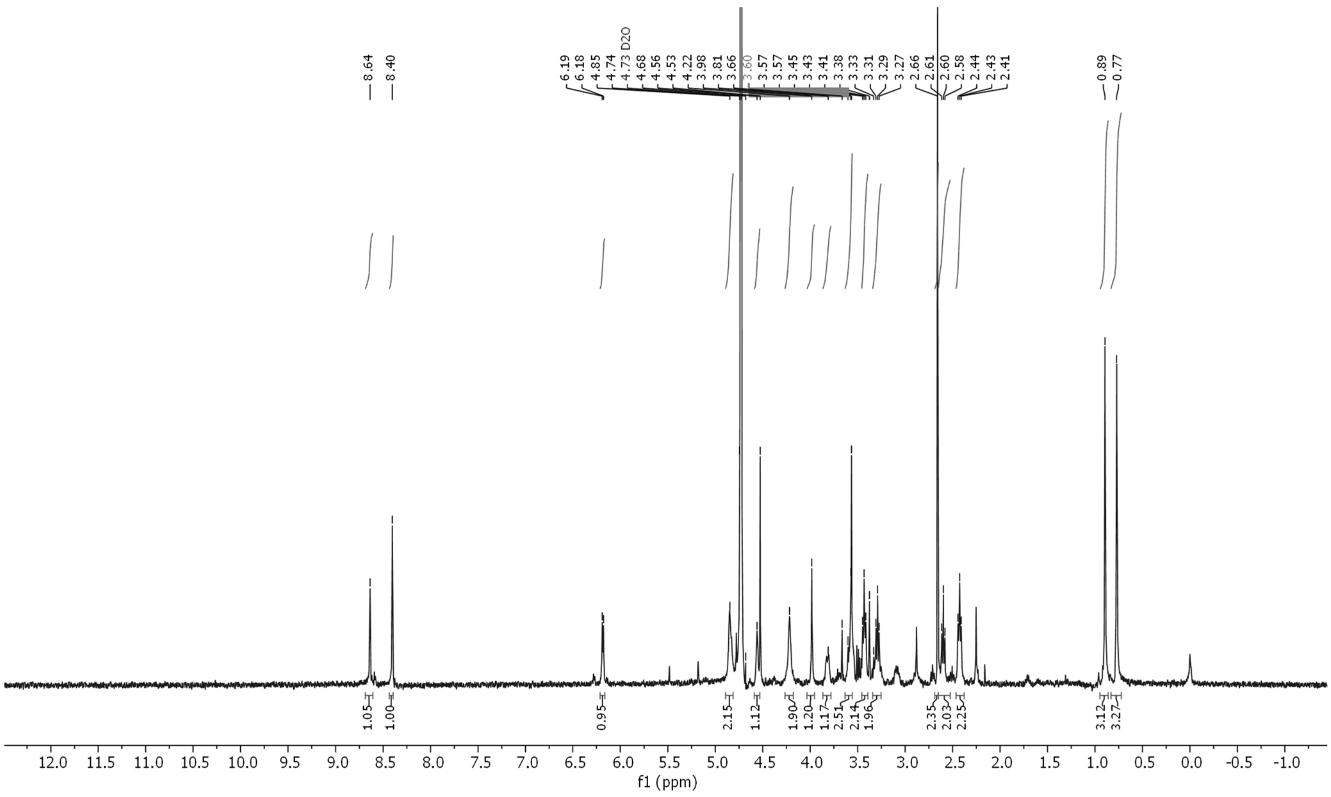

Figure S19. ^{1}H-NMR of **8**.

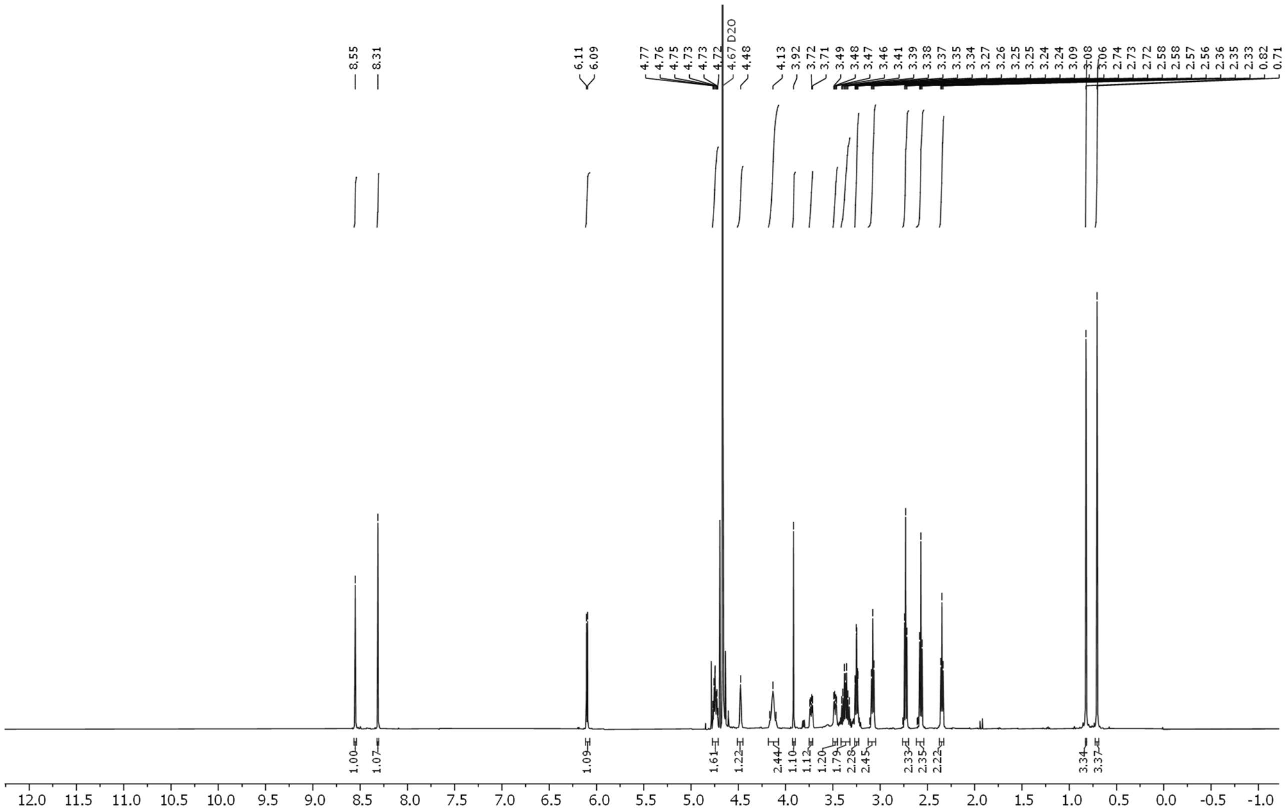

Figure S20. ^{1}H-NMR of **9**.

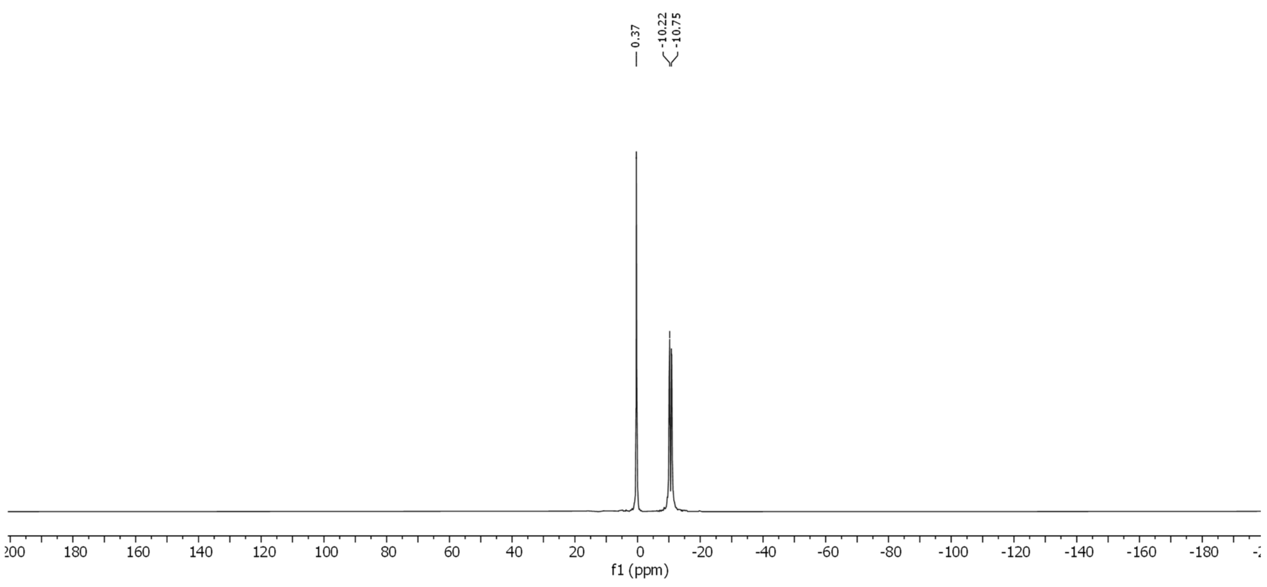

Figure S21. ^{31}P-NMR of **9**.

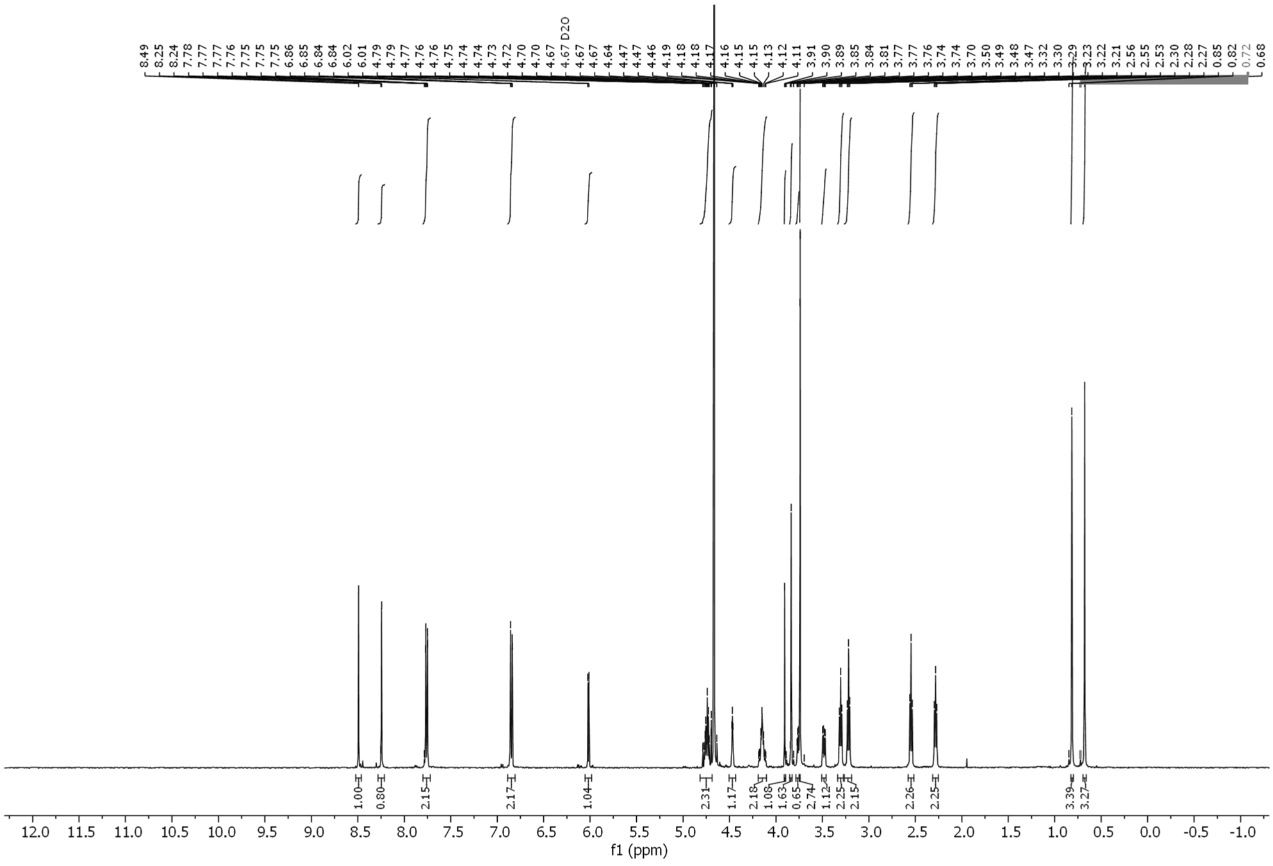

Figure S22. ^{1}H-NMR of **10**.

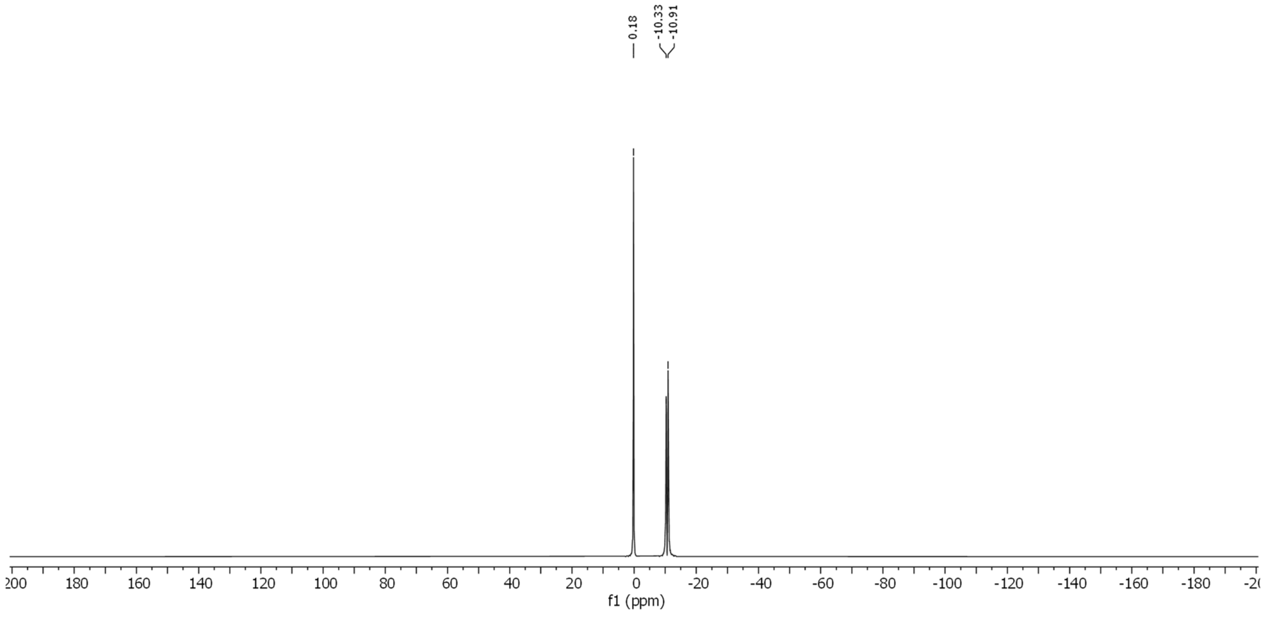

Figure S23. ^{31}P-NMR of **10**.

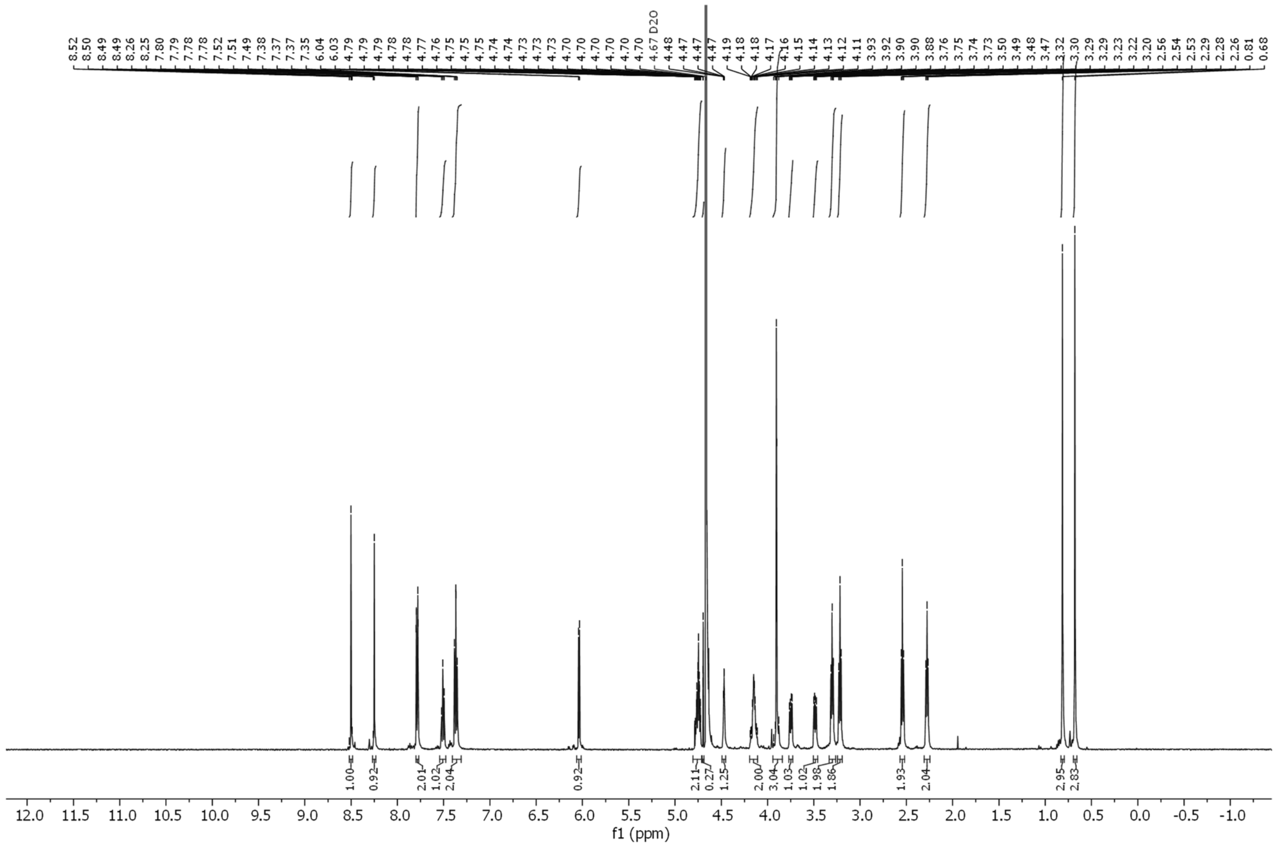

Figure S24. ^{1}H-NMR of **11**.

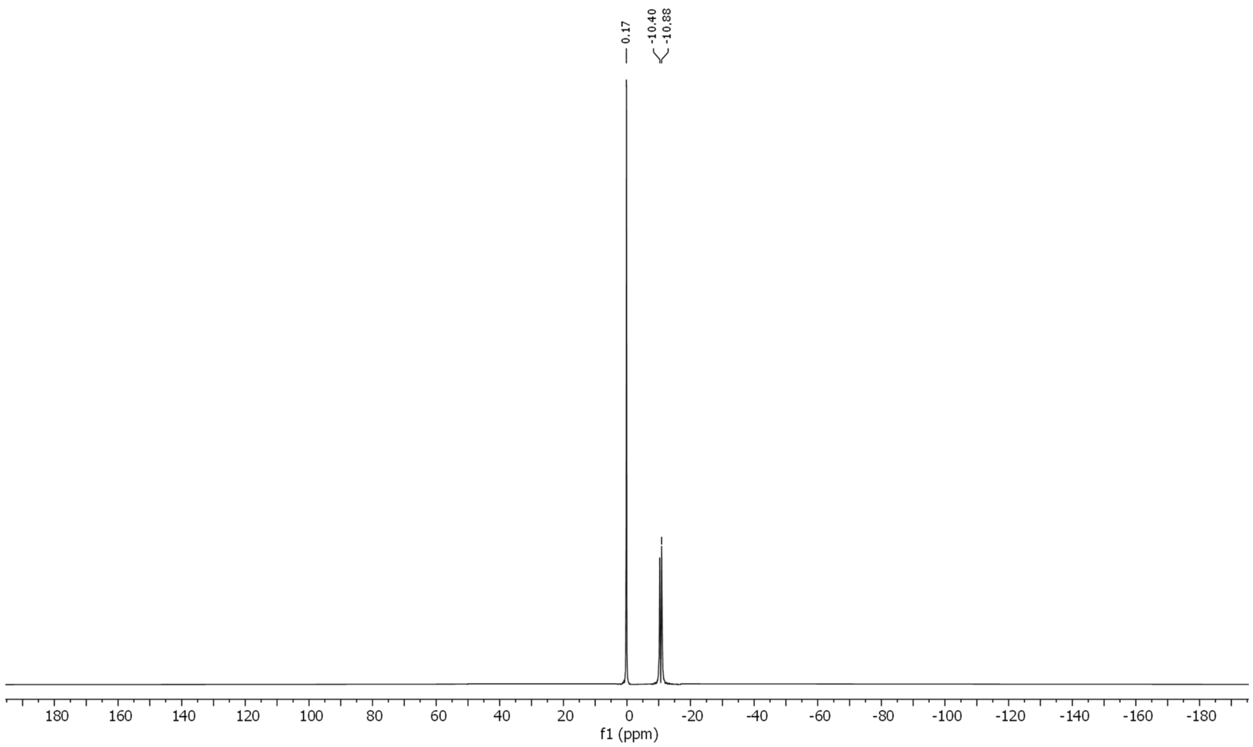

Figure S25. ^{31}P-NMR of **11**.

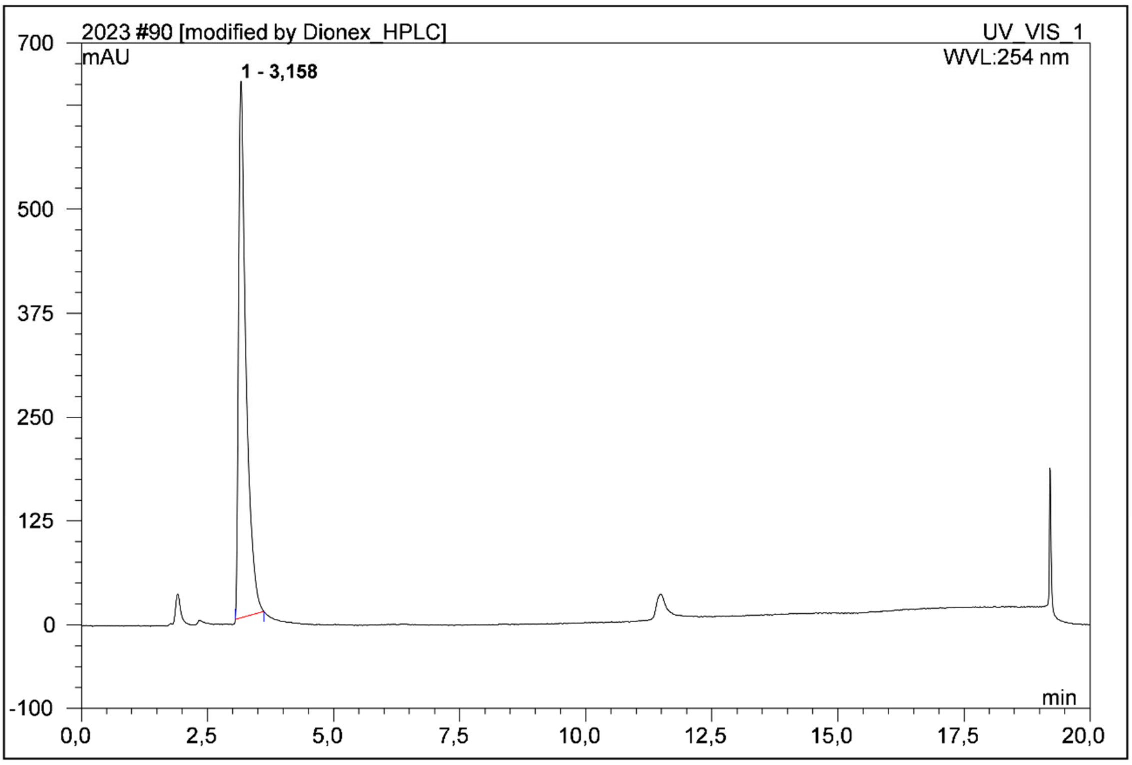

Figure S26. Analytical HPLC spectrum of **1**.

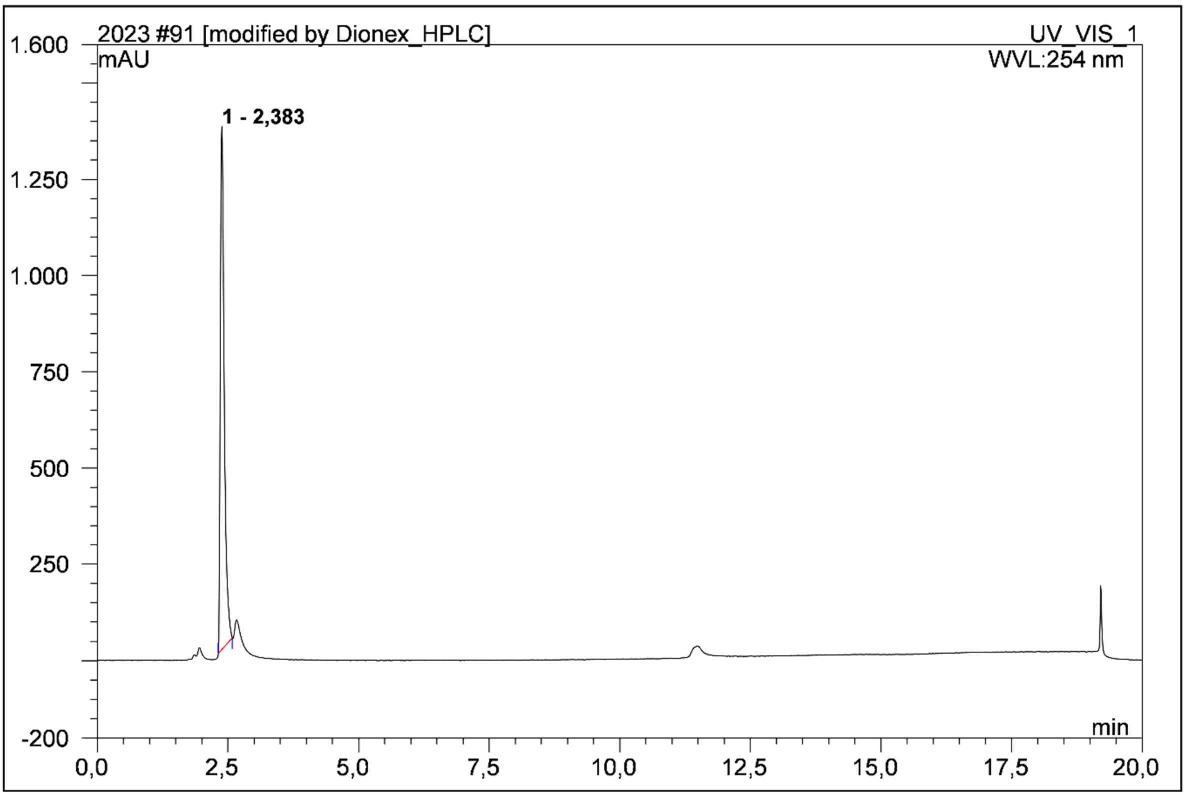

Figure S27. Analytical HPLC spectrum of **2**.

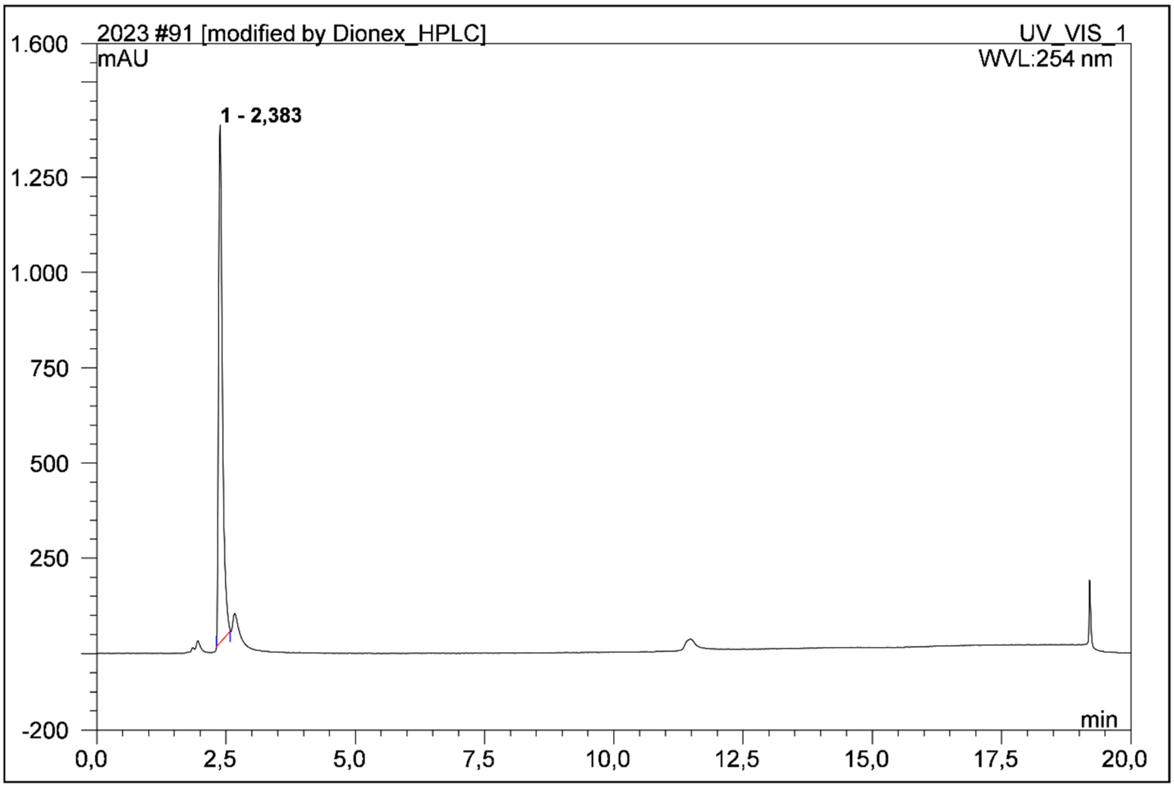

Figure S28. Analytical HPLC spectrum of **3**.

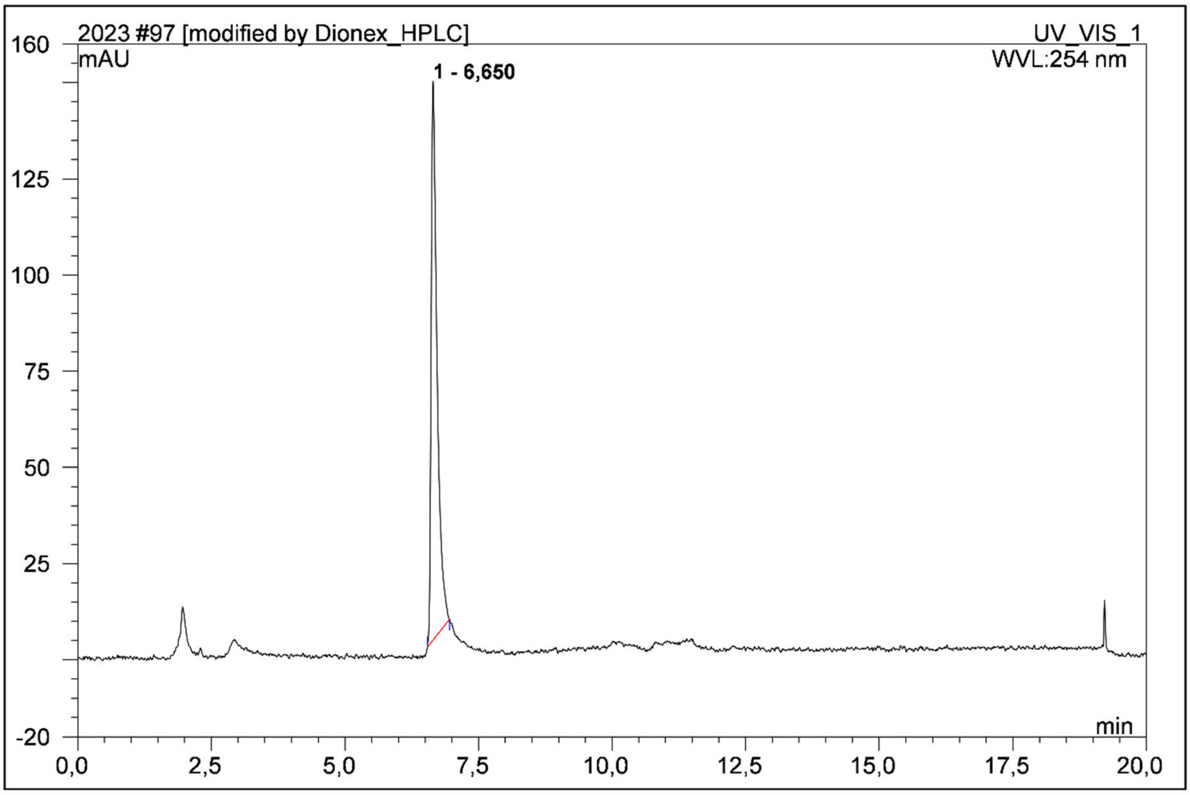

Figure S29. Analytical HPLC spectrum of **4**.

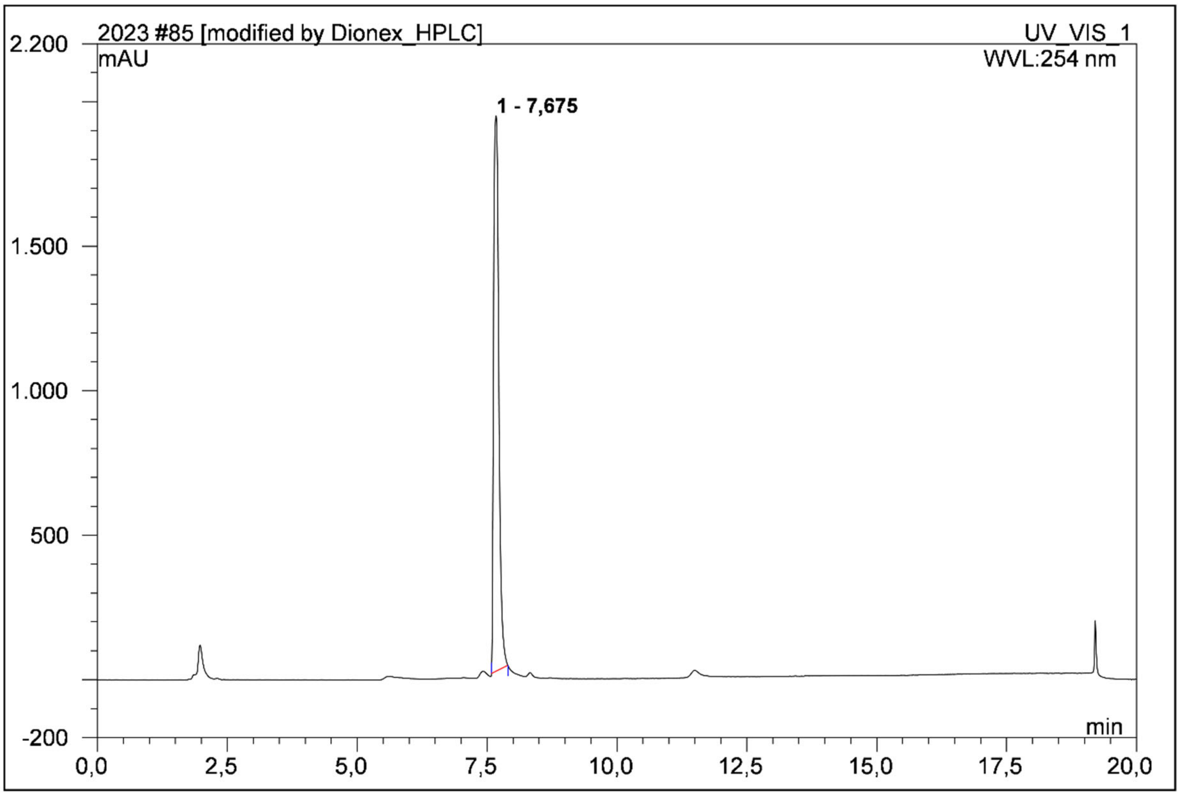

Figure S30. Analytical HPLC spectrum of **5**.

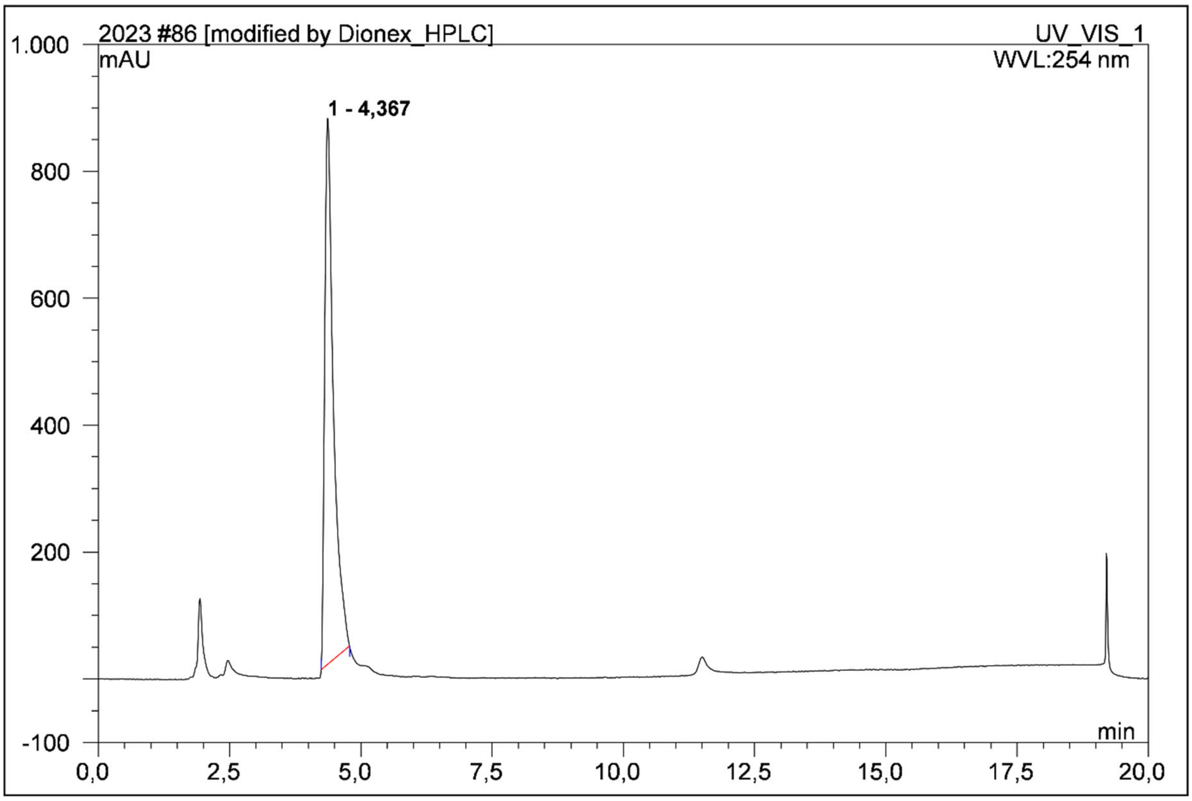

Figure S31. Analytical HPLC spectrum of **6**.

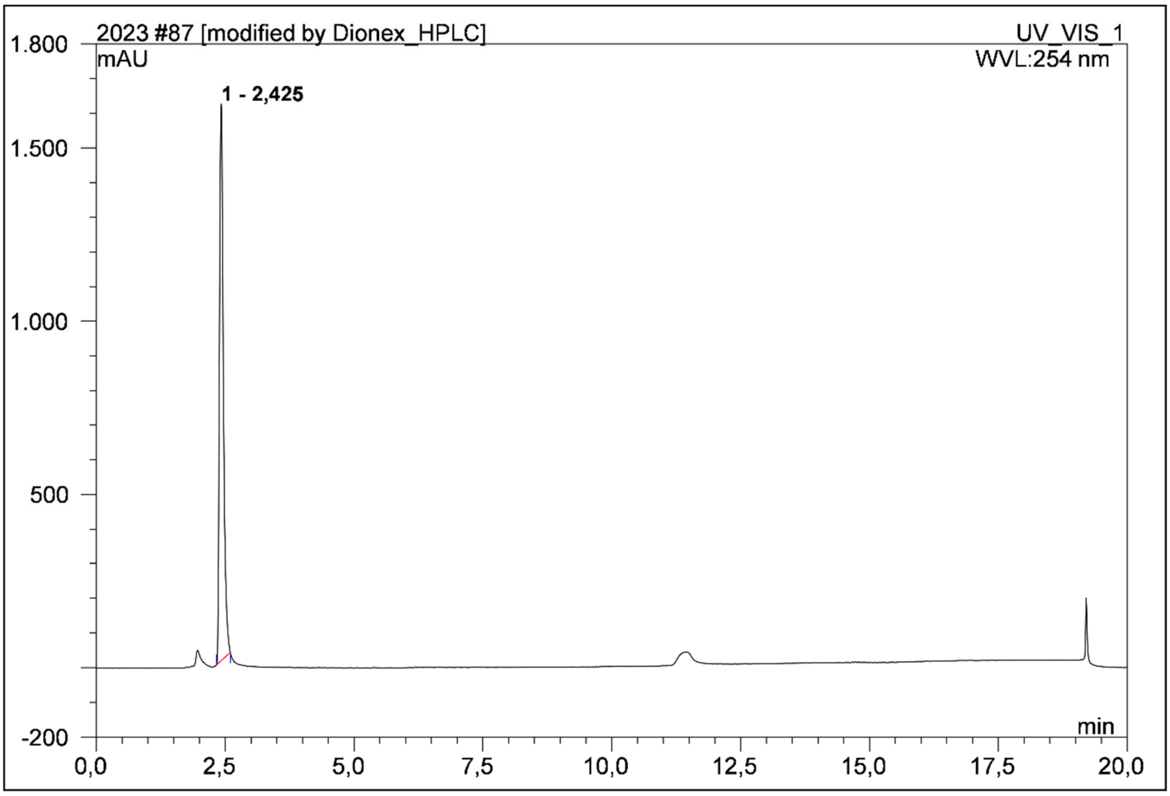

Figure S32. Analytical HPLC spectrum of **7**.

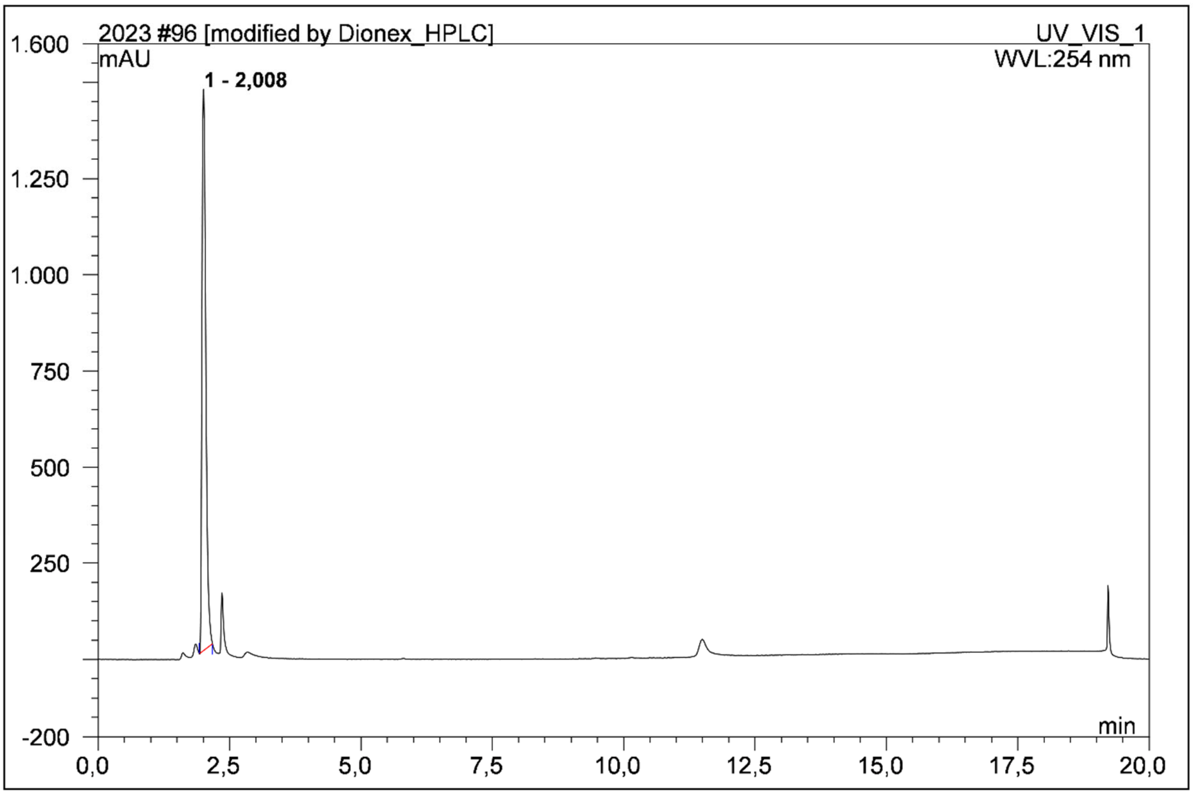

Figure S33. Analytical HPLC spectrum of **8**.

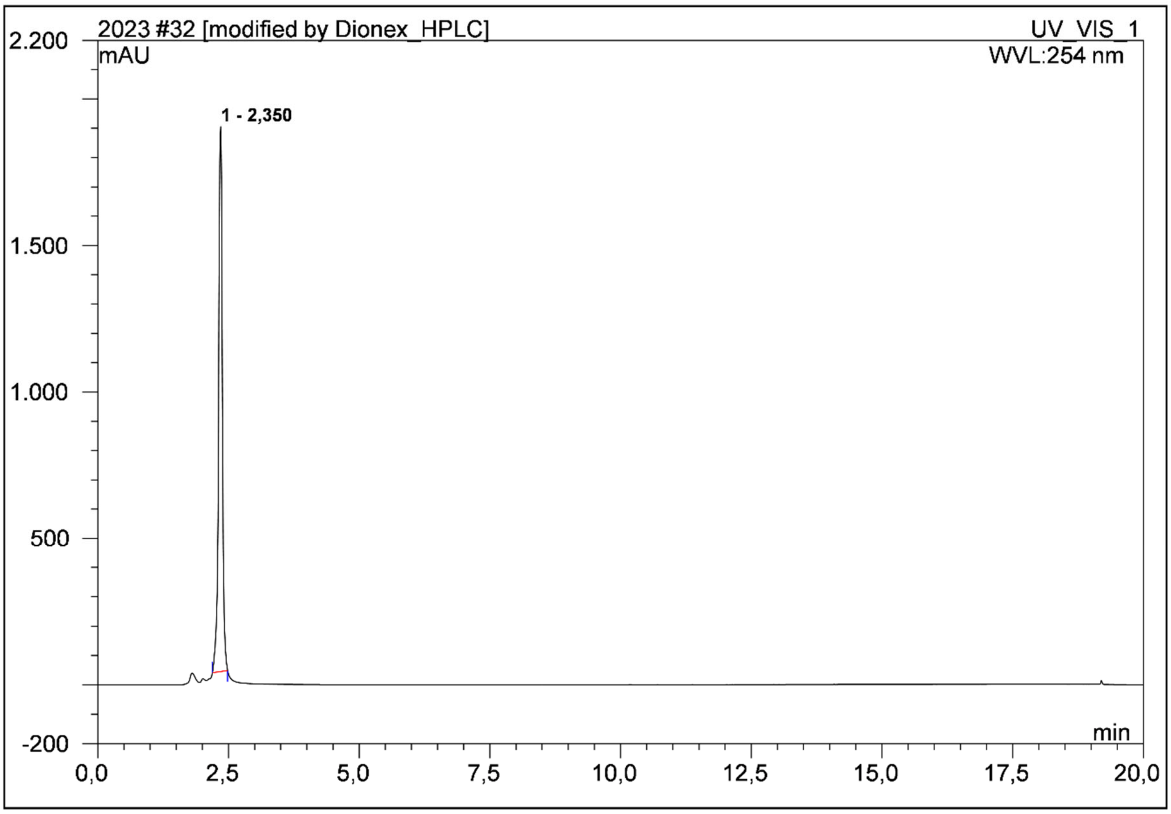

Figure S34. Analytical HPLC spectrum of **9**.

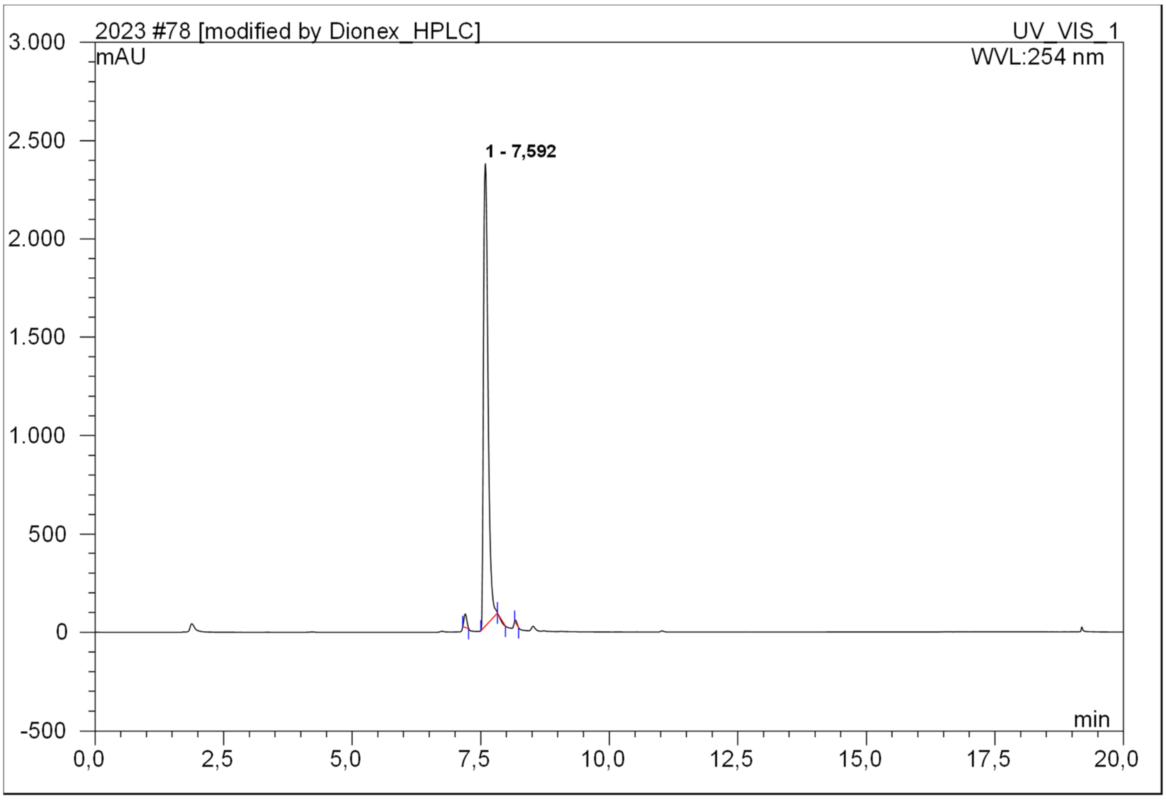

Figure S35. Analytical HPLC spectrum of **10**.

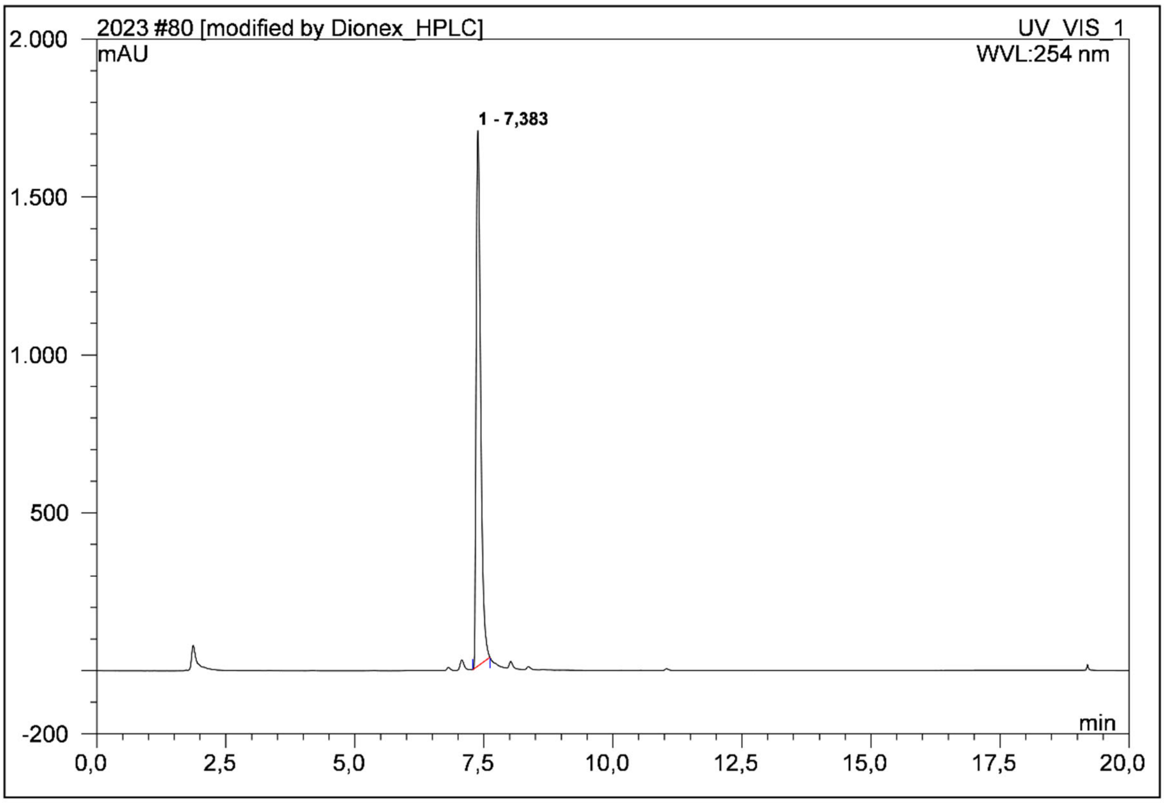

Figure S36. Analytical HPLC spectrum of **11**.
